# Biosimilar uptake policies in Europe: a cross-country mixed-methods policy analysis

**DOI:** 10.1016/j.lanepe.2026.101868

**Published:** 2026-10-01

**Authors:** Sabine Vogler, Katharina Habimana, Melanie Walter, Verena Knoll, AUGMENT Consortium, Elżbieta Kuriata, Katarzyna Leoszkiewicz, Kacper Mucha, Aleksandra Pelczarska, Elżbieta Skassa, Guenka Petrova, Petya Milushewa, Zornitsa Mitkova, Konstantin Tachkov, Julian Perelman, Joana Alves, Carolina Santos, Henrique Vasconcelos

**Affiliations:** a WHO Collaborating Centre for Pharmaceutical Pricing and Reimbursement Policies, Pharmacoeconomics Department, Gesundheit Österreich GmbH (Austrian National Public Health Institute), Stubenring 6, 1010 Vienna, Austria; b Department of Health Care Management, Technische Universität Berlin, Berlin, Germany

**Keywords:** Biological drug, Biosimilar, Uptake, Market share, Europe, Policy analysis, Effectiveness, Assessment

## Abstract

**Background:**

European countries implemented a range of policies to enhance uptake of biosimilar medicines, which are interchangeable with the originator but usually offer biological therapies at lower cost. However, challenges to biosimilar uptake persist. The study analysed the effectiveness of policies to improve biosimilar uptake.

**Methods:**

Based on 2024 IQVIA MIDAS® data, biosimilar market shares were determined for 27 European countries. Data on supply-side policies (i.e., targeting price and funding) and demand-side policies (i.e., addressing stakeholders) implemented across the countries was obtained from information generated by the Pharmaceutical Pricing and Reimbursement Information (PPRI) network and validated by country experts. Biosimilar market shares were analysed in relation to the policies implemented in each country. Selected case studies illustrated how biosimilar uptake developed before and after the introduction of a policy. A literature review identified evaluations of biosimilar policies implemented individually or as a package.

**Findings:**

Biosimilar market shares varied substantially across Europe, ranging from above 50% (e.g., Norway, Italy) to below 10% (Greece, Luxembourg). All countries studied had biosimilar policies in place, but their design varied. Frequent policies were tendering, encouraging prescribers to switch medication from the originator to the biosimilar, and price setting based on the originator's price (biosimilar price link). Gain-sharing models, switching, the biosimilar price link and prescribing quotas were associated with higher biosimilar market shares.

**Interpretation:**

The study confirmed the importance of implementing policies and suggested that demand-side measures are particularly effective in increasing biosimilar uptake.

**Funding:**

European Commission under the EU4Health Programme.

**Research in context**

**Evidence before this study**

We searched PubMed, Cochrane Library and EMBASE for articles written in English and published between Jan 1, 2000 and Sept 30, 2024, using the following search terms: ((biosimilar∗) OR (similar biological) OR (follow-up biological) OR (biologic∗)) AND (((policy) OR (pricing) OR (reimbursement)) OR ((tender∗) OR (price link∗) OR (education∗) OR (gainshar∗) OR (switch∗) OR (substitution) OR (reference pric∗) OR (anti-corruption) OR (International Nonproprietary Name prescribing) OR (guideline∗) OR (recommendation∗) OR (quota∗) OR ((prescribing) OR (prescription)) OR (competition) OR (negotiation∗) OR (auction∗)) AND ((benefit∗) OR (enabler∗) OR (advantage∗) OR (disadvantage∗) OR (barrier∗) OR (impact∗) OR (challeng∗) OR (facilitat∗) OR (assess∗) OR (evaluat∗) OR (best practice∗) OR (proven approach∗) OR (optimal method) OR (effective technique∗) OR (outcome∗) OR (encourage∗) OR (incentiv∗) OR (penalt∗))). After screening titles and abstracts of 6589 records and assessing the full texts of 444 records, we retrieved 82 studies from the search. The literature review conducted before this study was complemented by updates during the study, considering literature up to April 2026 (see Annex 3 in the Supplementary Materials). Several previous studies provided narrative information about the implementation of a biosimilar policy initiative in a country: commonly reported policy measures included tendering, switching, educational activities, and the biosimilar price link. Other studies mapped biosimilar policies implemented across high-income countries, including European countries; high-income countries with large markets were more frequently represented in the literature. Data on biosimilar use, including developments over time, was rarely linked to underlying policies, and the number of evaluation studies identified was rather limited. These studies examined the impact of a policy intervention, either implemented as a single policy or as combination of a few measures, on the uptake of biosimilars in a specific country or setting (e.g., in a hospital), as well as on other outcome indicators (e.g., savings). Prescribing quotas, gain-sharing models, switching and educational measures were found to have been effective in increasing the uptake of biosimilar medicines.

**Added value of this study**

This study brings together the three above-mentioned research topics (policy mapping, biosimilar uptake measurement, and policy assessment), by analysing both recent sales data and several biosimilar policies in 27 European countries. Cross-country variation in biosimilar uptake and in the design of policy measures despite frequent use of some policies (tendering, switching) was a key finding in this mixed-methods study. While existing literature typically tends to be focused on one single country or a small number of countries, and on a limited selection of biological active substances, this study stands out for its breadth of the countries covered and number of biosimilars included. Supplemented by country case studies on the developments in biosimilar uptake before and after a policy intervention, this study identified gain-sharing models and switching as policies associated with higher biosimilar market shares. These results confirm findings from the literature about the pronounced effectiveness of certain policies, particularly those in the demand side. Additionally, the study examined further biosimilar policies, whose effectiveness had not been studied before, even though these policies have meanwhile been implemented in several countries.

**Implications of all the available evidence**

The study underscores the importance of policy interventions to harness the benefits of biosimilar competition. It points to certain policies, particularly gain-sharing models and switching policies, as effective approaches to improve biosimilar uptake. Overall, fostering biosimilar uptake and competition appears to be more effectively achieved through demand-side policies. Introducing a biosimilar policy, policymakers are tasked with choosing the most appropriate methodological approach within the context of their country, since both the design of a policy and its interaction with other measures may influence the outcomes. Financial incentives, clear regulatory guidance, trust and capacity-building, as well as stakeholder information and engagement tend to play a supportive and reinforcing role in reaching intended policy objectives. The evidence generated in this study may encourage policymakers to implement biosimilar policies, which have proven effective in other countries. Once implemented, the policies should be monitored and assessed with regard to their impact and potential unintended consequences, and, if needed, be adapted. The implementation process, including the need for adjustments, will offer learnings to other countries.

## Introduction

Biological medicines offer important treatment options in key therapeutic areas, including oncology, immune-mediated inflammatory diseases, and endocrinology. However, originator biologicals tend to carry high price tags, which may challenge their affordability. After patent expiry and loss of further exclusivity rights of the originator, biosimilar medicines may be marketed. Biosimilars are developed to be similar to an existing biological medicine (i.e., their reference product) and have demonstrated comparable quality, safety, and efficacy to their reference product. Due to their lower prices, biosimilars can contribute to affordable access to biological therapies, and allow treatment of more patients even if the budget is not increased.^1^, ^2^, ^3^

Europe has a long history of approving and using biosimilars, where biosimilars undergo rigorous regulatory assessment by the European Medicines Agency before being permitted to be placed into the markets of the European Union (EU) and European Economic Area (EEA) member states. Europe is the leading region globally in terms of marketing authorisation for biosimilars,^4^ and biosimilar use in Europe was reported to surpass the rest of the world.^5^ Nevertheless, challenges to biosimilar uptake persist in Europe: While biosimilar market shares have increased over time, variation between countries, settings and substances was observed and points to untapped potential for uptake that has not yet been fully realised.^6^ Major barriers to biosimilar uptake include physicians' hesitancy towards biosimilar prescribing and switching due to skepticism about the biosimilar equivalence and immunogenicity concerns, patients’ fears regarding safety and efficacy, missing incentives for prescribing or dispensing biosimilars, and non-availability and delayed market entry of biosimilars in some countries due to patent litigation and “evergreening” strategies of the originator firms to delay entry of competing biosimilars.^1^^,^^3^^,^^5^^,^^7^, ^8^, ^9^

In response, European countries, whose governments are responsible for implementing policies in the peri-launch and post-launch phases (i.e., after marketing authorisation, which is centralised in the European Union), introduced policies targeting the price and public funding of biosimilars as well as measures to improve knowledge and acceptance of biosimilars among prescribers, pharmacists, and patients.^8^^,^^10^, ^11^, ^12^ The biosimilar policy landscape represents a dynamic area that is evolving, with some countries having adopted additional policies in recent years.

While the impact of policies to promote the uptake of generics has been well documented,^13^ evidence on the effectiveness of biosimilar policies is still limited. In this study, we mapped policies implemented to improve the uptake of biosimilars, investigated biosimilar uptake (measured as biosimilar market shares) across European countries, and explored how differences in biosimilar uptake may be associated with the policies implemented in the countries studied.

## Methods

The study employed a mixed-methods design, combining quantitative data analysis with the collation of policy information gathered from policymakers, and a review of relevant literature. High-level analysis of several policies surveyed across various countries was complemented by the study of specific policy interventions in some case study countries.

### Quantitative data analysis

IQVIA MIDAS® sales data were used for quantitative analysis. The IQVIA MIDAS® data sets that we obtained comprised all medicines that had recorded sales between 2008 and Q2/2024 in the countries covered by the data sets (including some non-EU and non-EFTA countries, see Annex 1). IQVIA MIDAS® sales data were provided for individual products (reference products and their biosimilars) at the level of manufacturer list prices, which may not reflect actual prices.

From the data sets, we extracted all biological substances, for which at least one biosimilar had recorded sales in at least one of the countries of our analysis, which were EU member states, European Free Trade Association (EFTA) countries, and the United Kingdom. This resulted in data of 27 active substances, comprising a total of approximately 200 products (reference products and biosimilars).

Although we initially aimed to examine biosimilar uptake across all EU and EFTA countries and the United Kingdom, we had to exclude the EU member states Cyprus, Denmark and Malta as well as EFTA countries Iceland and Liechtenstein, since no information was recorded for these countries in the IQVIA MIDAS® data sets. Thus, our analysis covered 27 European countries, including 24 of the 27 EU member states, two EFTA countries (Norway and Switzerland), and the United Kingdom.

Data from both the outpatient (i.e., community) and hospital settings were included in the analysis, except for Estonia, Greece, and Luxembourg, for which only data for the outpatient sector was reported.

For the year 2024 latest available data was from Q2/2024. For the case studies, we used respective longitudinal data to perform time-series analyses.

Some observations were excluded from the data sets where the specifications of eligible medicines could not be determined with sufficient reliability, due to missing data or apparently incorrect information (e.g., inconsistency between the reporting of strength and pack size information by IQVIA MIDAS® and corresponding information about authorised medicines by the European Medicines Agency). In cases where errors could be plausibly identified, corrective adjustments were made (e.g., reallocating information that appeared to have been reported in an incorrect field to the appropriate cell). Further information on the curation of the data set, including approaches to address related methodological challenges, can be found in Annex 1.

Overall biosimilar market shares for the countries studied were calculated as the total sales of all biosimilars across all biological substances divided by total combined sales of all biosimilars and their corresponding reference products of all included biological substances:

$$\text{Overall}\;\text{market}\;\text{share}=\frac{\sum_{j=1}^{m}\sum_{i=1}^{n_{j}}S_{ij}}{\sum_{j=1}^{m}\left(S_{j}^{R}+\sum_{i=1}^{n_{j}}S_{ij}\right)}\text{.}$$

whereby,

m is the total number of biological active substances included in the analysis,

$n_{j}$ is the number of biosimilars for active substance j,

$S_{ij\;}$ is the sales of the i-th biosimilar belonging to active substance j,

$S_{j}^{R}$ is the sales of the reference product for active substance j.

Using sales data as a proxy indicator for measuring biosimilar uptake is associated with limitations, which are further detailed in the discussion section.

### Policy mapping

Guided by biosimilar policy overviews in the literature,^8^^,^^11^^,^^12^^,^^14^ we developed a taxonomy to classify policies adopted for biosimilars, either to impact their price and funding (supply-side policy measures) or to target key stakeholders, including those who prescribe, dispense and use medicines (demand-side measures, Table 1). While pricing, procurement and reimbursement policies are typical peri-launch policies (i.e., implemented after marketing authorisation and usually before the market entry of a product), most demand-side measures are taken after market entry (post-launch policies). However, educational and awareness-raising activities can also be performed at other points across the life-cycle of a medicine. Horizon scanning is a pre-launch policy action.

**Table 1:** Taxonomy of biosimilar policies.

Policy domain	Policy	Definition
**Supply-side policies**		
Pricing & procurement	Biosimilar price link	Practice of setting the price of a biosimilar medicine, in relation to the price of the reference product (originator biological), usually at a defined percentage rate lower than the reference product price.
	External price referencing for biosimilars	Practice of using the price(s) of a biosimilar in one or several countries in order to derive a benchmark or reference price for the purposes of setting or negotiating the price of the product in a given country.
	Tendering	Any formal and competitive procurement procedure through which tenders (offers) are requested, received and evaluated for the procurement of goods, works or services, and as a consequence of which an award is made to the tenderer whose tender/offer is the most advantageous.
	Tendering-like practices	Pricing and procurement policies which include competitive components (e.g., tenders for medicines of the same active ingredient and selection of one or a few for inclusion in reimbursement), typically applied by public payers (e.g., social health insurance funds) in the outpatient setting.
Reimbursement	Inclusion of biosimilars in a reference price system	A reimbursement policy in which identical medicines (same substance) or similar medicines (same pharmacological or therapeutic group) are clustered (reference group). The public payer funds a maximum amount (the reference price), while the patient must pay the difference between the reference price and the pharmacy retail price of the medicine, in addition to any co-payments (such as prescription fees or percentage co-payment rates). Reference price systems may be designed differently, e.g., on whether the inclusion of biological medicines is allowed.
System preparedness	Horizon scanning on loss of exclusivity	While horizon scanning in general relates to the systematic identification of health technologies that are new, emerging or becoming obsolete and that have the potential to affect health, health services and/or society, the policy applied in the biosimilar context aims to inform in advance about the loss of exclusivity of originator biologicals and thus support public payers and procurers to be well prepared to harness the benefits of biosimilars from day zero.
**Demand-side policies**		
Targeting doctors	Prescribing by the International Non-Proprietary Name (INN) for biologicals	Requirement for prescribers (e.g., physicians) to prescribe a biological medicine by its International Non-Proprietary Name/INN, i.e., the active ingredient name instead of the brand name. INN prescribing may be allowed (indicative INN prescribing) or required (mandatory/obligatory INN prescribing).
	Switching	Action by a prescriber to change medication (e.g., from an originator biological to a biosimilar medicine or from one biosimilar to another biosimilar medicine).
	Prescribing quotas	Requirement to physicians and further prescribers to achieve defined thresholds in prescribing, typically determined shares of biosimilar prescribing for defined active substances or therapeutic groups.
	Educational and awareness-raising activities	Action by governments and their institutions, health care providers and further stakeholders to inform doctors and potential further prescribers about biosimilar medicines and related relevant aspects. This includes dissemination of clinical evidence on biosimilar medicines and the immunogenicity concept to address wide-spread misperceptions (such as the assumption of increased risk of immunological reactions after a switch). These activities may also inform about regulatory and policy provisions and guidance documents as well as build capacity regarding prescribing biosimilars, and related communication to patients. Educational and awareness-raising activities may be linked to other demand-side measures targeting prescribers, e.g., providing guidance for switching medication.
	Gain-sharing	Models which offer (portions of) savings (e.g., resulting from biosimilar use) to health care institutions (e.g., hospitals) or health professionals (e.g., doctors), typically earmarked for further investments in health care.
Targeting pharmacists	Biosimilar substitution	Practice of dispensing a biosimilar medicine instead of the prescribed equivalent and interchangeable biosimilar or biological originator medicine at the pharmacy level without consulting the prescriber. In some countries also referred to as automatic biosimilar substitution.
	Gain-sharing	Models which offer (portions of) savings (e.g., resulting from biosimilar use) to health care institutions or health professionals (e.g., pharmacies, pharmacists), typically earmarked for further investments in health care.
	Educational and awareness-raising activities	Action by governments and their institutions, health care providers and further stakeholders to inform pharmacists about biosimilar medicines and related relevant aspects. This includes dissemination of clinical evidence on biosimilar medicines and the immunogenicity concept to address wide-spread misperceptions. These activities may also inform about regulatory and policy provisions and guidance documents and build capacity regarding dispensing of biosimilars, and related communication to patients. Educational and awareness-raising activities may be linked to other demand-side measures targeting pharmacists, e.g., providing guidance for biosimilar substitution.
Targeting patients	Gain-sharing	Models which offer (portions of) savings (e.g., resulting from biosimilar use) to patients.
	Educational and awareness-raising activities	Action by governments and their institutions, health care providers and further stakeholders to inform patients about biosimilar medicines and related relevant aspects. This includes dissemination of clinical evidence on biosimilar medicines and the immunogenicity concept to address wide-spread misperceptions, as well as information about regulatory and policy provisions. Overall, they aim to increase patients' confidence in biosimilars.

Information on the implementation status of the policies as of 2024 was predominantly sourced from the Pharmaceutical Pricing and Reimbursement Information (PPRI) network. PPRI is a global network of public authorities that work on pharmaceutical pricing and reimbursement policies and covers all 27 countries of this study.^15^ Through their involvement in PPRI, the authors had access to unpublished information generated within PPRI, including data on major country policies relating to biologicals and biosimilars.

To systematically collect policy information, we developed a standardised country fiche (Annex 2). In November 2024, the fiche template was piloted with two countries (Denmark, Norway). Afterwards, we completed it for all countries using data accessible through PPRI. We invited officials from public authorities that were members in the PPRI network to validate the information for their respective countries and to supplement it where necessary. 21 country fiches were validated (unvalidated country fiches from Croatia, Hungary, Ireland, Latvia, Luxembourg, and the Netherlands).

### Supplementary literature reviews

To place the results within the context of existing evidence, we conducted a series of literature reviews to identify studies that examined the impact of policies on biosimilar uptake: First, we conducted a systematic review of studies published in English language in the peer-reviewed literature, with data extraction completed in October 2024. This was complemented by narrative reviews undertaken in the preparation of the country fiches, which also considered studies in English and local languages in both the peer-reviewed and grey literature. We also considered any other eligible literature published until April 2026 that reported the effects of policies on biosimilar uptake. These additional studies were identified through monitoring of new publications, snowballing techniques, and recommendations provided by validators and reviewers. Annex 3 provides further details of the methodology of the literature review, including the search strategy.

### Case studies

The study was enriched by an analysis of four case studies on country policy changes: switching guidance from the regulatory medicines agency in Italy, prescribing quotas in France, and the introduction of biosimilar substitution in Norway and France. Two considerations guided the selection of the case studies: we were especially interested in policies which appeared to be particularly effective (findings from the literature and own analysis), and in policies that had been implemented more recently, thus less represented in the policy analysis literature. We analysed the evolution of the market shares of the biological substances targeted by the policy measures, covering the period from 2008 (where applicable) until Q2/2024 (latest available data).

### Role of the funding source

The study was commissioned to the AUGMENT Consortium through a tender under the EU4Health Programme by the European Commission. In compliance with the Tender Specifications, the Consortium was required to use IQVIA MIDAS® data, which had been purchased by the European Commission and provided to Consortium members under a Third Party Agreement. Officials from the European Commission reviewed the study's methodology and a draft of a technical study report that forms the basis of this article. Beyond these contractually defined oversight activities, the funder of the study had no role in the study design, in the collection, analysis, and interpretation of data, in the writing of the report, and in the decision to submit the paper for publication.

## Results

### Biosimilar uptake across European countries

Biosimilar market shares in Europe increased substantially over the years, reaching on average 40.7% across all countries studied (Q2/2024), compared to 3.6% in 2014 (lowest value: 0.1% in Luxembourg, highest value: 8.6% in Poland). However, large variation persisted across the countries. Whereas biosimilar market shares in Norway, Italy, Croatia, the United Kingdom, and Finland exceeded 50%, uptake remained below 10% in Greece and Luxembourg, where data was only available for the outpatient sector (Fig. 1).

**Fig. 1:**
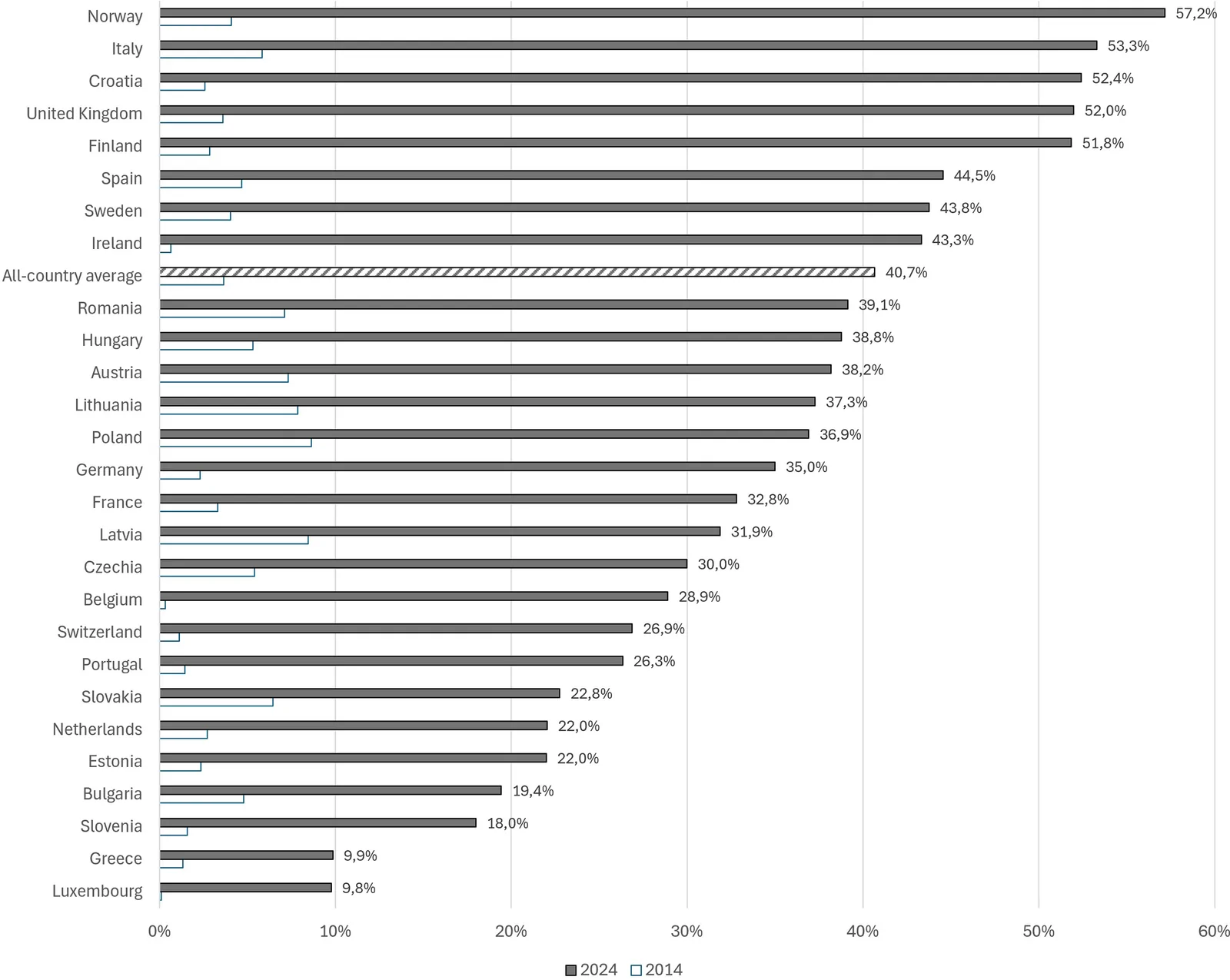
**Biosimilar market shares (in sales), in 27 European countries, in 2024 compared to 2014.** Data for Estonia, Greece and Luxembourg reflect only the outpatient (community) sector and do not include the inpatient (hospital) sector. Biosimilar market shares were calculated as percentage of total market shares in sales and are indicated in per cent (unweighted average). 2024 data cover only Q1 and Q2/2024. Authors’ analysis based on IQVIA MIDAS® sales data for Q1/2008-Q2/2024 reflecting estimates of real-world activity. Copyright IQVIA. All rights reserved.

### Biosimilar policies across European countries

Several policies relating to biosimilars have been implemented across European countries (Table 2 and Annex 4). In all countries studied, biosimilars, particularly those used in the hospital setting, are subject to tendering procedures. Further frequently implemented supply-side policies include the biosimilar price link (21 countries) and the inclusion of biologicals into the reference price system (18 countries). On the demand side, almost all analysed countries (24 in total) encourage, or even mandate, prescribers to switch patients from a biological originator to a biosimilar, or among biosimilars. In 17 countries prescribers are allowed to prescribe biologicals by the International Non-Proprietary Name (INN), and 15 countries apply prescribing quotas for biosimilars. Biosimilar substitution at pharmacy level is yet less common (11 countries), though a few countries introduced it after 2020 (Norway in 2021, France in 2022, and Finland, Germany and Switzerland in 2024). In some countries, substitution targeted solely few biological substances initially, with the aim of gradual expansion. Gain-sharing models were identified in solely ten countries. The models exclusively target prescribers and are typically limited to a single health care setting (most often hospitals, e.g., the Netherlands, United Kingdom), region (e.g., in Italy) or payer (e.g., initiatives by social health insurance institutions in Germany). No gain-sharing models that targeted pharmacists or patients were identified.

**Table 2:** Biosimilar policies in place in 27 European countries 2024.

Policy	Biosimilar price link	EPR for biosimilars	Tendering^a^	Tendering-like prac^a^	RPS incl. biosimilars	Horizon scanning on LoE^b^	INN prescr. for biologicals	Switching^c^	Prescribing quotas	Gain-sharing^d^	Biosimilar substitution
Austria	Yes	No	Yes	No	No	Yes	No	Yes	No	No	No
Belgium	Yes	No	Yes	No	Yes	Yes	Yes	Yes	Yes	Yes	No
Bulgaria	Yes	Yes	Yes	No	Yes	No	Yes	No	No	No	Yes
Croatia	Yes	Yes	Yes	No	Yes	No	Yes	Yes	Yes	No	No
Czechia	Yes	Yes	Yes	Yes	Yes	No	Yes	Yes	No	No	Yes
Estonia	Yes	No	Yes	No	Yes	Yes	No	Yes	No	No	Yes
Finland	Yes	Yes	Yes	No	Yes	No	Yes	Yes	No	No	Yes^f^
France	Yes	Yes	Yes	No	No	Yes	No	Yes	Yes	Yes	Yes
Germany	No	No	Yes	Yes	Yes	No	No	Yes	Yes	Yes	Yes^g^
Greece	No	Yes	Yes	No	Yes	No	No	Yes	Yes	No	No
Hungary	Yes	Yes	Yes	Yes	Yes	Yes	Yes	Yes	Yes	Yes	No
Ireland	Yes	No	Yes	No	No	Yes	Yes	Yes	No	No^e^	No
Italy	Yes	No	Yes	Yes	No	Yes	Yes	Yes	Yes	Yes	No
Latvia	Yes	No	Yes	No	Yes	No	Yes	Yes	Yes	No	Yes
Lithuania	Yes	Yes	Yes	No	Yes	Yes	Yes	Yes	No	No	No
Luxembourg	No	Yes	Yes	No	No	No	No	–	–	No	No
Netherlands	No	No	Yes	Yes	Yes	Yes	Yes	Yes	Yes	Yes	Yes
Norway	Yes	Yes	Yes	No	Yes	Yes	Yes	Yes	No	No	Yes
Poland	Yes	Yes	Yes	No	Yes	Yes	Yes	Yes	No	No	Yes
Portugal	Yes	Yes	Yes	No	No	Yes	Yes	Yes	Yes	Yes	No
Romania	Yes	Yes	Yes	Yes	Yes	No	No	Yes	Yes	No	No
Slovakia	Yes	Yes	Yes	Yes	Yes	No	Yes	Yes	Yes	No	No
Slovenia	Yes	Yes	Yes	No	Yes	No	Yes	Yes	No	No	No
Spain	Yes	Yes	Yes	No	Yes	Yes	No	Yes	Yes	Yes	No
Sweden	No	No	Yes	No	No	Yes	No	Yes	Yes	Yes	No
Switzerland	Yes	No	Yes	No	No	Yes	Yes	No	No	No	Yes^f^
UK	No	No	Yes	No	No	Yes	No	Yes	Yes	Yes	No

Definitions of the policies are provided in Table 1. Educational and awareness-raising activities, mostly provided by regulatory agencies and the biosimilar industry, but also clinically-scientific societies, public payers and public procurers, were identified in all countries; however, they were not included because their scope and content varied considerably, which limited comparability. Policies may be implemented in different designs. Further information on some specifications of the design is provided in Annex 4. Information on the implementation of the biosimilar policies, and design characteristics is sourced from the Pharmaceutical Pricing and Reimbursement Information (PPRI) policy network of public authorities responsible for pharmaceutical pricing and reimbursement and validated by experts.

EPR: external price referencing. INN: International Non-Proprietary Name. LoE: loss of exclusivity. RPS: reference price system, which builds clusters for biologicals. prac.: practice. presc.: prescribing. UK: United Kingdom.

a While tendering rather relates to tendering procedures in the hospital sector, tendering-like practices, usually carried out by reimbursement authorities and public payers, such as social health insurance institutions, tend to be applied in the outpatient setting.

b Done systematically or at least occasionally.

c “Yes” relates to a situation with a policy in place that obliges, encourages or supports switching to a biosimilar by prescribers (e.g., regulatory guidance document), “no” indicates that no such policy was identified.

d Solely information on gain-sharing models incentivizing biosimilar prescribing; no gain-sharing models for dispensing biosimilars (pharmacists) or for asking for biosimilars (patients) were identified.

e No gain sharing in 2024, but some pilots in earlier times.

f Implemented in January 2024.

g Implemented in March 2024.

### Effectiveness of policies

Examining individual policies in a descriptive cross-sectional analysis, biosimilar market shares were found to be, on average, highest in countries that had introduced gain-sharing models, encouraged prescribers to switch medication to biosimilars, applied a biosimilar price link and imposed prescribing quotas. In addition, tendering-like procedures of public payers for medicines used in the outpatient sector, thereby granting preferential reimbursement and/or market access to suppliers, and, more generally, tendering, as commonly applied in hospitals, were also associated with higher biosimilar market shares. Some of the policies, which were linked to higher biosimilar shares, were implemented in only a limited number of countries which tended to have larger markets (Fig. 2).

**Fig. 2:**
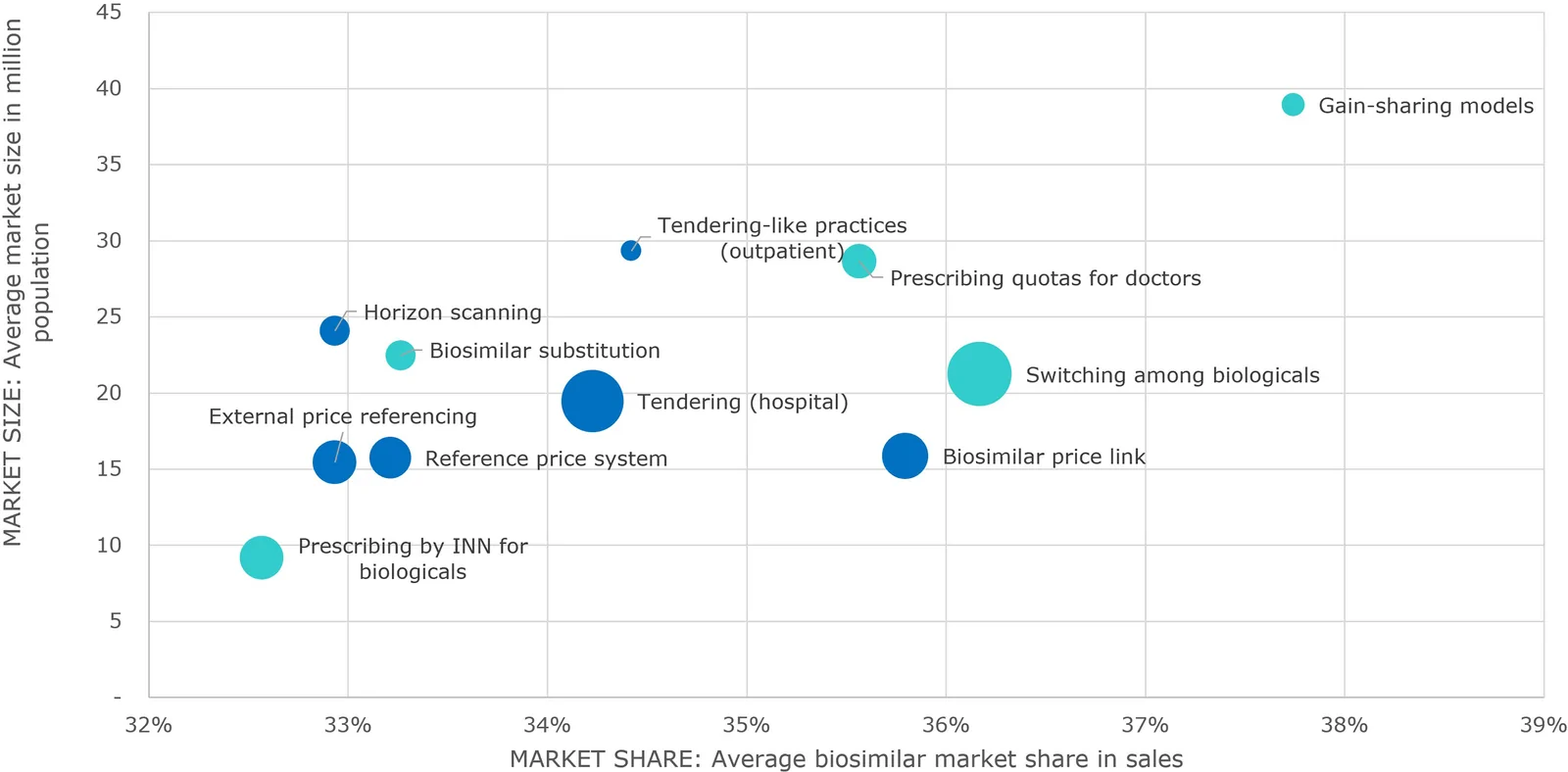
**Average biosimilar market shares of countries having implemented specific policies, and their market size, 2024 data.** Market size is measured by population data sourced from Eurostat (https://ec.europa.eu/eurostat/databrowser/view/tps00001/default/table?lang=en&category=t_demo.t_demo_pop). Biosimilar market shares were calculated as percentage of total market shares in sales based on IQVIA MIDAS® sales data for Q1/2008-Q2/2024 reflecting estimates of real-world activity. Copyright IQVIA. All rights reserved. Data for Estonia, Greece and Luxembourg reflect only the outpatient (community) sector and do not include the inpatient (hospital) sector. Blue circles: supply-side biosimilar policies; green circles: demand-side biosimilar policies. The size of the circles indicates the number of countries that have implemented a specific policy (e.g., gain-sharing has a small circle as applied in rather few countries, whereas tendering, which is in place in all countries, has a large circle). Exploratory descriptive analysis, which should not be misinterpreted as presenting causal relationships.

In four illustrative case studies, the development of biosimilar market shares was analysed over a defined period, during which a specific biosimilar policy was introduced. Across all four case studies, the introduction of the analysed policies (switching, prescribing quotas, and biosimilar substitution) was followed by an increase in the market shares of the targeted biosimilars, however to different extents (Fig. 3).

**Fig. 3:**
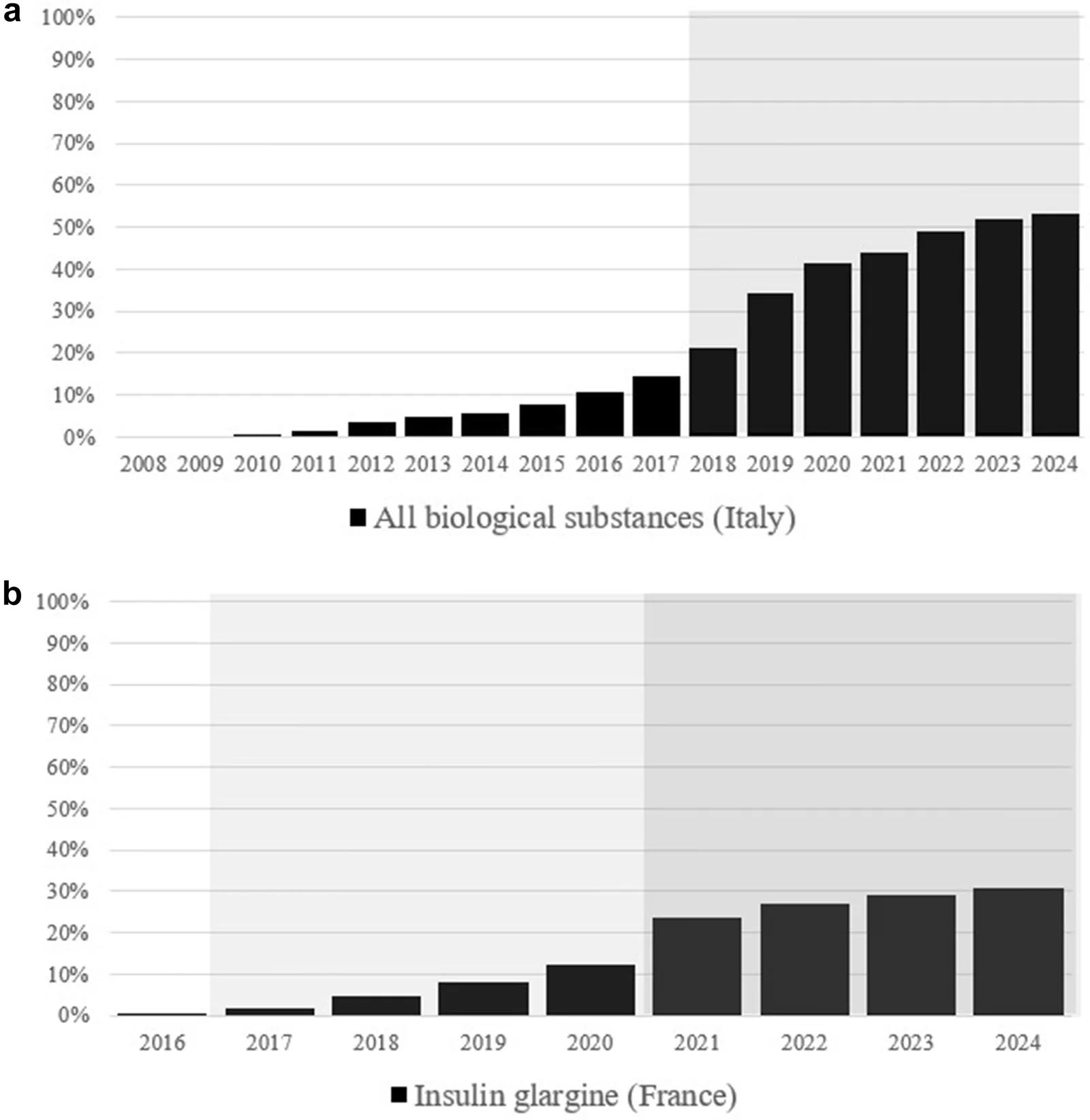

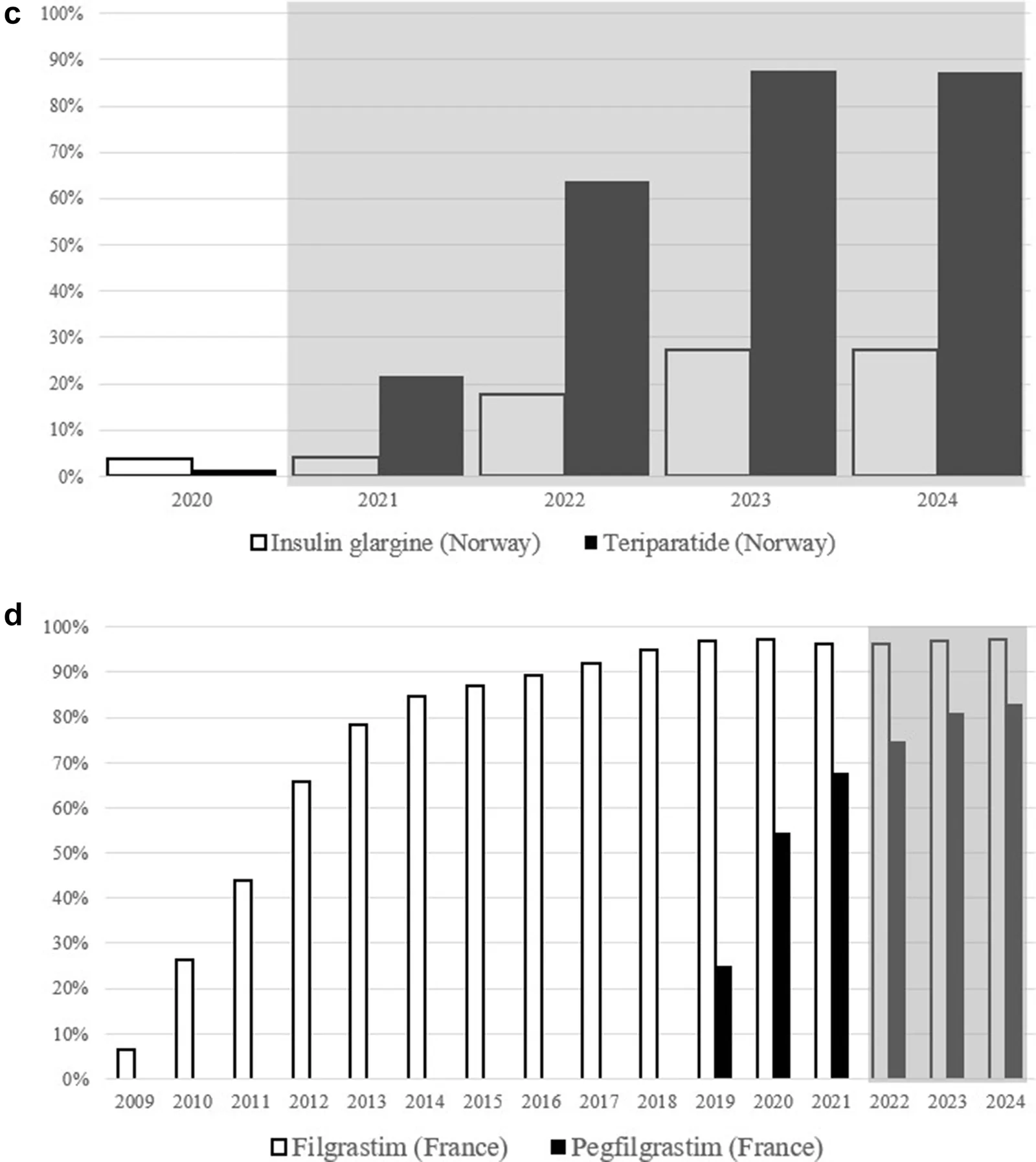
**Evolution of biosimilar market shares before and after a policy intervention in four case studies.** Biosimilar market shares in sales, of either all biological substances, as included in the analysis (A) or specific substances subject to the policy intervention studied (B, C, D). Biosimilar market shares were calculated as percentage of total market shares in sales and are indicated in per cent. 2024 data cover only Q1 and Q2/2024. (A). Supportive switching policy in Italy: publication of a position paper on the interchangeability of biological medicines by the Medicines Agency in March 2018, which endorses prescriber-initiated switches from biological originators to biosimilars and among biosimilars. (B). France: prescribing quotas of 20% were introduced for insulin glargine at the beginning of 2017 and increased to 40% in 2021. (C). Biosimilar substitution was introduced in Norway in a step-wise approach, starting with insulin glargine and teriparatide subject to biosimilar substitution from November 2021. (D). Biosimilar substitution was introduced in France in a step-wise approach, starting with filgrastim and pegfilgrastim subject to biosimilar substitution from April 2022. Authors’ analysis based on IQVIA MIDAS® sales data for Q1/2008–Q2/2024 reflecting estimates of real-world activity. Copyright IQVIA. All rights reserved.

In addition, empirical evaluations in the literature that explored the impact of certain policies on biosimilar uptake (16 studies identified) pointed to prescribing quotas, gain-sharing models and further financial incentives, switching policies, communication activities and tendering as policies that proved effective in increasing biosimilar uptake (Table 3). One study reported that, while the introduction of the biosimilar price link reduced prices, it had no impact on biosimilar uptake. In several instances, policy interventions consisted of a combination of measures whose individual effects were not assessed. Annex 5 provides additional details on the reviewed studies. Findings from the literature underscore the importance of an appropriate policy design, and highlight the role of clearly defined policy objectives, preparedness, active communication and stakeholder engagement as supportive elements for improving biosimilar uptake.

**Table 3:** Empirical evidence in the literature on the impact of policies on biosimilar uptake.

Policy/policies	Country/countries	Outcome	Study
Individual policy			
Biosimilar price link	Hungary	Biosimilar uptake did not increase	Harsanyi et al. 2020^16^
Tendering-like system	Hungary	Biosimilar uptake increased	Hornyák et al. 2019^17^
Encouraging switching	United Kingdom	Biosimilar uptake increased	Barron et al. 2023^18^
Prescribing quotas	Germany	Biosimilar uptake increased	Moorkens et al. 2020^19^
	Italy (Campania region)	Biosimilar uptake increased initially and then became stable	Menditto et al. 2015^20^
Gain-sharing	France	Biosimilar prescribing and uptake increased	Tano et al. 2023^21^ Tano et al. 2024^22^
	France	Biosimilar uptake increased, savings were generated	Attia et al. 2024^23^
	Ireland	Biosimilar uptake increased, savings were generated	Duggan et al. 2021^24^
Mix of policies			
Switching guidelines, communication to healthcare professionals and patients, and tendering	Denmark	Biosimilar uptake increased	Jensen et al., 2020a^25^ Jensen et al., 2020b^26^
Prescribing quotas, inclusion in reference price system and design of pricing policies	Germany	Biosimilar uptake increased in most cases, whereas uptake of the reference product decreased	Oppel et al. 2025^27^
Prescribing quotas, financial incentive, monitoring on adequate hospital tendering, education campaign (two waves of policy interventions)	Belgium	Mixed findings: uptake increased but to different extents (partially low) across the substances and the different policies	Vandenplas et al. 2023^28^
Mandatory regional tendering, communication, and financial incentive	Italy	Biosimilar uptake increased more substantially in the region with the policy intervention compared to other regions	Giudotti et al. 2021^29^
Single policies across countries			
Tendering, biosimilar price link, and prescribing quotas	13 countries (including 9 European countries)	Biosimilar uptake appeared to have increased due to these three policies; but only tendering apparently reduced expenditure	Tam et al. 2025^14^
Nine policies (5 demand-side policies, 4 supply-side policies)	8 countries (including 6 European countries)	Demand-side policies, such as financial incentives and prescribing guidelines, are significantly associated with higher biosimilar uptake	Shin et al. 2025^30^

## Discussion

The study provides updated data on biosimilar market shares, and confirmed persisting substantial variation across European countries, as shown in earlier analysis.^6^ In addition, by offering more recent information on biosimilar policies across several European countries, it highlighted the developments in biosimilar policies over the last years, when countries introduced additional biosimilar policy measures (e.g., switching, biosimilar substitution, financial incentives). Finally, by analysing both sales data and policy information for a large number of European countries and in further detail for selected case studies, the study identified policies which were associated with higher biosimilar market shares, thus suggesting their effectiveness in driving biosimilar uptake. As such, this research adds to earlier studies that applied similar methods.^14^^,^^30^ Previous research was often restricted to a limited selection of biological active substances and a single country or a small number of countries. This study benefits from the breadth of the countries and biological active substances included.

The financial incentive policy of gain-sharing models is particularly distinctive in being associated with higher biosimilar market shares. At the same time, gain-sharing models were implemented in only few countries, mostly countries of higher income and with large markets (e.g., France, Germany, United Kingdom). Findings in the literature were consistent with our results. Moreover, the literature review stressed the importance of an appropriate methodological design, to ensure that those who contributed (e.g., respective wards) got acknowledged rather than overall sharing gains with the hospitals.^21^^,^^22^

Another policy associated with increased biosimilar uptake is switching by prescribers. Since prescribers tend to show continued hesitance to switch clinically stable patients to a biosimilar, clear guidance communicated by regulatory agencies, and further authorities (e.g., payers), targeted at prescribers was found to play an essential role. This was also illustrated by the Italian case study, which showed increases in biosimilar market shares after the medicines agency had published a regulatory guidance document on the safety of switching.

Regulatory guidance benefits from switching studies, as their results from the real-world settings have served to inform and thus build capacity and trust among prescribers, who may become more confident in their communication with patients. The findings of switching studies on the safety of a switch are also important to challenge the narrative about the risk of biosimilar switching (e.g., regarding immunogenicity concerns), which continues to persist. It has even been argued in the literature that given the accumulated evidence on biosimilar safety after switching, improving communication should be prioritised over conducting additional switching studies.^31^ The value of communication was also underscored by an increase in infliximab biosimilar prescribing in Norway upon informal availability of draft findings of the seminal NOR-SWITCH study, which confirmed the safety of that switch, although the study had not yet officially been published.^32^

Quantitative analysis identified prescribing quotas (i.e., asking doctors to prescribe defined biosimilar shares for certain biological substances) as another important biosimilar policy. This was supported by findings from a case study on France and the literature.^27^^,^^28^

Our findings confirm previous research which suggested that demand-side policies, which aim to address the behaviour of stakeholders responsible for prescribing (doctors), dispensing (pharmacists) or using biosimilars (patients) would be more effective in increasing biosimilar uptake compared to supply-side policies.^19^^,^^30^ Since key barriers to biosimilar uptake lie in stakeholder reservations towards biosimilars, including hesitancy of doctors to switch, well-designed demand-side measures may provide leverage.

However, biosimilar substitution in pharmacy, though another policy on the demand side, was not associated with comparably higher biosimilar market shares in our analysis. A possible explanation could be the relatively recency of this policy in some countries (Finland, Germany and Switzerland introduced it in 2024) and its sometimes gradual implementation, starting with a limited set of defined biological substances (e.g., Finland, France, Norway). It is likely too early for determining measurable effects. Nonetheless, the two case studies on biosimilar substitution suggested a positive effect, as the uptake of those biosimilars subject to substitution increased rapidly after its implementation (unless in case of already high biosimilar market shares).

Among the supply-side policies, the biosimilar price link and tendering were associated with higher biosimilar market shares compared to other policies. For those policies, design is also essential. For instance, a study concluded that the decision to prescribe a biosimilar may also be influenced by the size of the price difference between the originator biological and the biosimilar, which should be sufficiently large to trigger change.^27^

Targeting the price, supply-side policies rather generate savings for the health system, which is a key element to contribute to affordable patient access to medicines. While there is a large body of evidence on both savings generated through biosimilars and the untapped savings potential,^9^ tendering continues to be viewed as a controversial policy: concerns have been voiced that it may drive down prices to levels which may risk making markets unattractive for suppliers so that no biosimilars, or generics, would be offered.^9^ Such potential availability issues can be mitigated through “strategic procurement” which implies consideration of further award criteria than the price, granting of contracts to multiple winners and more centralised or collaborative organisation of procurement, as some best practices from both generic as well biosimilar tendering indicate.^25^^,^^26^^,^^33^ It has been shown that strategic procurement, which contributes to patient access through affordable prices without availability issues, goes even beyond the pure tendering process: it encompasses application of horizon scanning, health technology assessment, and engagement with relevant actors, including health care professionals, patients and suppliers.^33^ Successful switches in Danish hospitals underscore the importance of the different components.^25^^,^^26^

In this respect, the role of anti-trust enforcement is also key, where competition authorities monitor, and also take action, against originator companies which, as part of their evergreening strategies, impose anti-competitive conditions on procurers to deter biosimilar competitors.^33^

While the quantitative analysis conducted in this study examined single policies, in practice effects on uptake are likely attributable to a combination of policies, and other external market related factors. No conclusive findings were obtained regarding the role of the market size in influencing biosimilar uptake. While some large countries reported higher biosimilar market shares, this pattern was not consistent across all large countries. At the same time, some smaller countries implemented several biosimilar policies, including measures that were associated with increased biosimilar uptake. A higher number of policies may result in improved biosimilar uptake, as an intra-country comparison of two settings with variation in policy implementation showed, while acknowledging further influencing factors.^27^

Some evaluations studied a policy package implemented rather than a single policy and concluded that implementation of a carefully designed mix of measures has proven instrumental in achieving the intended policy objectives.^25^^,^^26^^,^^29^

In addition to the amplification of individual effects into broader joint outcomes through the combination of relevant policies, the design of the policies is key. The literature review conducted for this study confirmed the positive impact of accompanying information and engagement activities, not only as stand-alone action such as a campaign but also as a component built into the policies studied (e.g., information activities to prescribers in Norwegian and Danish hospitals on procured biosimilars). The degree of stakeholder involvement associated with policy implementation varies across countries and policy intervention, ranging from one-way provision of information to more interactive and participatory formats. The literature on biosimilar policies confirmed the intuitive assumption that stronger levels of engagement were effective in changing biosimilar-related behaviour of relevant stakeholders such as clinicians, further health care professionals, patients, as well as hospital managers and administrators.^18^^,^^25^^,^^26^^,^^29^

This study has some limitations. The design for the quantitative analysis was constrained by the scope and content of the IQVIA MIDAS® data set. We acknowledge that, by using sales data, consumption (uptake) measurement was influenced by monetary components (i.e., the prices of the medicines included). In addition, sales data were measured at manufacturer list price level, which may not reflect actual prices that may be lower after negotiation and procurement processes. Taking sales data as a proxy for consumption is associated with limitations and constitutes a weakness of the study. The gold standard in drug utilisation research would be consumption measurement by Defined Daily Doses.

Furthermore, although the authors would have had access to 2025 policy information for all EU and EFTA member states, the quantitative analysis had to be restricted to 27 countries since no IQVIA MIDAS® data was available for Denmark, Cyprus, Iceland, Liechtenstein, and Malta, and the data set only covered information up to Q2/2024. The analysis of these small countries would have been interesting, also for examining the assumption that smaller markets may be underserved. Denmark would have been an interesting case to study, since the literature^8^^,^^11^^,^^25^^,^^26^ and PPRI information indicated implementation of several biosimilar policies. Furthermore, the effects of more recently introduced policies in some countries, such as biosimilar substitution, could not be fully captured within the available data period.

For this exploratory research, policies were assessed according to whether they were in place or not, but no design specifications were considered in the quantitative analysis. The study disregarded a potential reinforcing effect of policy combinations, since the consequences of individual measures as part of a policy package are difficult to disentangle. No formal correlation or regression analysis was conducted. Consequently, the study does not claim any causal effect of individual policies, or combination of policies, but aims to illustrate patterns in policy implementation and their association with observed biosimilar market shares.

Differences in biosimilar market shares across countries may also be caused by other factors, including the date of marketing authorisation. However, we consider this bias rather low due to centralised regulatory approval of biosimilars in EU and EEA member countries (25 countries of the study). It mainly concerns Switzerland which had considerably fewer biosimilars approved compared to the other study countries, whereas the United Kingdom, an EU member state before 2020, figured similar approval rates as EU countries.^4^

Future research could be devoted to the construction of a multi-variate composite indicator to measure the contribution of specific biosimilar policies on the uptake of biosimilars as well as on other access components, such as affordability, typically expressed in savings generated through biosimilars, or acceptability on behalf of patients. A methodology to examine the impact of specific design elements of biosimilar policies could be developed and piloted. Moreover, policies in the pre-launch domain, such as facilitating and potentially accelerating regulatory processes, e.g., around clinical trials, that were beyond the scope of this study, could also be another field for future research.

### Conclusion

The study highlighted the dynamic nature of both biosimilar policy implementation and biosimilar markets across several European countries. In light of the next wave of biosimilars expected to enter the markets in the coming years and which will challenge high-priced originator biologicals, policymakers are encouraged to harness the benefits of biosimilar competition and to exploit untapped potential by fostering biosimilar uptake. While effective policy interventions often consist of a combination of policies, ideally combining demand-side and supply-side measures, the study indicates that certain demand-side policies are particularly effective in improving biosimilar uptake: these include financial incentives such as gain-sharing models, as well as policies to steer prescribing behaviour, including switching to biosimilars. Since empirical evidence of more recently introduced policies remains limited, further data collection and analysis are essential to support continuous learning and strengthening the policy design as countries continue to implement and refine policies related to biosimilars.

## Contributors

Sabine Vogler: Conceptualization, Methodology, Validation, Formal analysis, Investigation, Writing—Original Draft, Writing—Review & Editing, Visualization, Supervision, Project administration, Funding acquisition. Katharina Habimana: Conceptualization, Methodology, Formal analysis, Investigation, Writing—Review & Editing, Visualization, Funding acquisition. Verena Knoll: Investigation, Writing—Review & Editing, Visualization. Melanie Walter: Methodology, Formal analysis, Investigation, Writing—Review & Editing, Visualization.

## Data sharing statement

The underlying MIDAS data were provided by IQVIA under a Third Party Agreement. The terms of our agreement do not permit disclosure, sublicensing, or sharing of IQVIA MIDAS data. IQVIA will honour legitimate requests for MIDAS data from qualified researchers. Please contact IQVIA to seek approval for data access; a licence fee might be applied.

## Declaration of interests

All authors declare that the study received funding from the European Health and Digital Executive Agency (HaDEA) acting under the mandate from the European Commission under Contract No. HADEA/2023/OP/0040 (Capacity building to support the uptake of biosimilars in a multistakeholder approach), awarded to the Austrian National Public Health Institute.

## Appendix A Supplementary data

Annex
