# Supplementary material for: Biosimilar uptake policies in Europe: a cross-country mixed-methods policy analysis
Source: Lancet Reg Health Eur. 2026 Oct 1;70:101868. doi: 10.1016/j.lanepe.2026.101868 (PMC13659503; doi:10.1016/j.lanepe.2026.101868)
Supplement: Supplementary file 1 [file mmc1.pdf]

Biosimilar uptake policies in Europe: a cross-country mixed-methods policy analysis

Supplementary Materials

Table of Content

Annex 1: Curation of the data for the quantitative analysis……………………………..……………….2

Annex 2: Country fiche template – review of policies…………………………………………………4

Annex 3: Methodology of the literature reviews and identified studies…………………………………..11

Annex 4: Design specifications of biosimilar policies…………………………………………………14

Annex 5: Description of pieces in the literature that evaluated impact of biosimilar policies on biosimilar uptake………………………………………………………………………………………………18

Annex 1: Curation of the data for the quantitative analysis

The primary source for quantitative data was IQVIA MIDAS® data, as has been specified in the Tender Specifications by the European Commission that provided funding for this study. The European Commission had purchased the IQVIA MIDAS® data, and based on respective Third Party Agreements, members of the Consortium were granted access to data extracted from IQVIA MIDAS®.

The data sets

These data comprised monthly, quarterly and for some exceptions, annual value and volume sales data for 27 European countries from Q1/2008 to Q2/2024. Included countries were Austria, Belgium, Bulgaria, Croatia, Czechia, Estonia, Finland, France, Germany, Greece, Hungary, Ireland, Italy, Latvia, Lithuania, Luxembourg, Netherlands, Norway, Poland, Portugal, Romania, Slovakia, Slovenia, Spain, Sweden, Switzerland and United Kingdom, as well as countries beyond the scope of the study (Belarus, Kazakhstan, Serbia, Russia, Türkiye, and the United States). Since IQVIA did neither record nor own sales data from Cyprus, Denmark, Iceland, Liechtenstein and Malta, these five European Union (EU) / European Free Trade Association (EFTA) countries were not included. For Estonia, Greece, and Luxembourg, data was available only for the outpatient sector. The following data disclaimer is applicable: "IQVIA market research information is proprietary to IQVIA and available on a confidential basis by subscription from IQVIA. IQVIA market research information reflects estimates of marketplace activity and should be interpreted accordingly.".

Data was provided by IQVIA in two separate files: One file contained data from Q1/2008 to Q3/2020, provided in three formats: quarterly intervals, yearly intervals, and monthly intervals, and another file covered the period from Q3/2012 to Q2/2024, also presented in quarterly, yearly, and monthly intervals.

Generating a comprehensive dataset for the analysis

To ensure consistency in timeline and enable cross-country comparisons, it was necessary to merge the files provided. However, merging the files posed challenges due to overlapping time periods and differing interval formats. While the files overlapped in terms of years, they did not fully align in terms of content for the defined set of active substances for the analysis. In some cases, identical product presentations (e.g., specific pack sizes) showed discrepancies in data for the same periods.

For biological substances within the scope, File 1 contained 13,960 usable rows, and File 2 contained 16,717. Of these, 7,447 rows matched in terms of pack description variables (excluding volume and value data). However, when comparing the actual numeric data (sales volumes and values) for these packs in both datasets, none of the rows were identical across both datasets.

Analysing the data, we assumed that differences in currency conversion (e.g., United States dollars / Euros vs. local currency) and potential inflation adjustments at the time of data extraction may have contributed to these discrepancies. Even after excluding sales value variables from the comparison, 3,470 rows still differed between the datasets. An extract highlighting these discrepancies, specifically the differences in units between the two files, was shared with IQVIA for clarification.

IQVIA investigated and attributed some of the differences to back data changes that had affected the IQVIA MIDAS® database between the two data deliveries. Notably, hospital data for Bulgaria and Italy were impacted. These local changes were acknowledged to have significant effects on global data. IQVIA recommended using the most recent data and rerunning analyses where necessary. In addition to back data changes, IQVIA noted that global updates, such as changes in classification systems (e.g., Anatomical Therapeutic Chemical (ATC) Classification and New Form Codes (NFC) codes via annual European pharmaceutical market research association (EphMRA) or World Health Organization (WHO) updates) and patent information revisions, could also affect the dataset. Given these potential changes, it was advised to limit data extraction to only the attributes required for the analysis.

To mitigate the methodological issues, it was decided to use data from the first file only up to Q2/2012 and from the second file from Q3/2012 onwards.

Curation and enrichment of the dataset

We excluded data from countries outside the study scope (such as Belarus, Kazakhstan, Serbia, Russia, Türkiye, and the United States). Regarding the data frequency, only quarterly data was retained, allowing for the calculation of annual aggregates.

In terms of medicines, all non-biological medicines were excluded. Additionally, biological medicines without a biosimilar available on the market in 2024 (or with no biosimilar expected in the near future) were also removed from the dataset. The data set then included 27 biological active substances (ADALIMUMAB, AFLIBERCEPT, AGALSIDASE ALFA, AGALSIDASE BETA, BEVACIZUMAB, DENOSUMAB, ECULIZUMAB, ENOXAPARIN SODIUM, EPOETINS, ETANERCEPT, FILGRASTIM, FOLLITROPIN ALFA, INFLIXIMAB, INSULIN ASPART, INSULIN GLARGINE, INSULIN LISPRO, NATALIZUMAB, OMALIZUMAB, PALOPEGTERIPARATIDE, PEGFILGRASTIM, RANIBIZUMAB, RITUXIMAB, SOMATROPIN, TERIPARATIDE, TOCILIZUMAB, TRASTUZUMAB, USTEKINUMAB), comprising approximately 200 medicines (reference products and biosimilars).

We curated the data set including exclusion of certain data entries from the IQVIA files to ensure analytical accuracy and consistency. In several instances, the calculation of the total amount of active substances per package could not be reliably derived from the provided IQVIA fields as these did not correspond to the actual strength, volume, or pack size authorised in the included countries. Corrections were made when the total amount of active substances was clearly inconsistent (e.g., the description contained other units than mg, or there was only one strength registered). In cases where these fields were missing, the amount of active substance was derived using other fields in the data set.

In addition to the data provided by IQVIA, the comprehensive dataset was enriched with supplementary information not explicitly included in the original files.

To enable categorisation by product type, the products within scope were first classified as reference products, biosimilar products, and one hybrid product. Subsequently, anonymised identifiers were assigned to each product based on the number of products available on the European market per active substance.

The anonymisation of international trade names, as indicated in the IQVIA data set, into acronyms per active pharmaceutical ingredient (API) was conducted using the European Medicines Agency (EMA) marketing authorisation list (as of 24 January 2025), supplemented by manual searches for international trade names. A directory file was created to match the IQVIA data with information from the EMA list.

Sales data was provided for the individual products, at the level of manufacturer list prices, and calculated for the categorised groups of biosimilars and reference products (see above). The biosimilar market shares (in sales) were calculated as the share of biosimilar sales of total sales of all included active biological substances.

For the data extraction, python 3.12 and R statistical computing software (version 4.4.1; R Core Team 2024) were used. The calculations and development of the plots were done in Excel for Microsoft 365.

Annex 2: Country fiche template – review of policies

The country fiche template included several sections, since the policy review was one of the research tasks of the AUGMENT Biosimilars study. In the following, solely the section of the policy framework regarding biosimilars will be presented.

Further sections of the country fiche template included:

- Introductory section, informing about the project, including the policy reviews, and providing practical instructions (e.g., timelines, contact details),
- Consent and acknowledgements of the validators,
- Narrative summary of key information of all sections of the country fiche,
- Key data (publicly accessible data sources relating to biosimilars in the respective country, e.g., biosimilars marketed, market shares),
- Stakeholders involved in and targeted by biosimilar policies (listing key public authorities and further stakeholder groups),
- Collation of relevant studies about the impact of the country's policies on biosimilar uptake, savings and further outcomes,
- Barriers in biosimilar implementation in the country,
- Facilitators and good practice examples in the country,
- Developments in the country,
- Further relevant literature with regard to biosimilars in the country, and
- Glossary of key terms.

The policy framework section listed several supply-side and demand-side policies to provide a comprehensive picture of the country.

Biosimilar policies and practices in country

Below, information on different biosimilar policies and practices is provided (and in some cases, also on further medicines, e.g., on generics or other biosimilars – presented in the grey shaded lines in the tables). Note that the information relates to the year 2024, to the national level and to outpatient and inpatient sectors, unless specified differently.

Is the information presented further below correct? Are there any features that should be further stressed? Please make changes directly in the text, using either track changes mode or, alternatively, red font, and use the space below each table to provide additional information, if required. Remember that for key terms highlighted in blue (note: this was done in the original template, but highlights were removed for the article), definitions are provided in the AUGMENT Biosimilars Glossary.

4.1 Pricing and procurement policies 2024

Biosimilar price link

Definition: Practice of setting the price of a biosimilar medicine, in relation to the price of the reference product (originator biological), usually at a defined percentage rate lower than the reference product price.

A **biosimilar price link** is in place.	Yes / no
It applies for the following situations:	For biosimilar medicines in the outpatient and inpatient sectors entering the market / For biosimilar medicines in the outpatient sector entering the market / For biosimilar medicines in the inpatient sector entering the market / For biosimilar medicines aiming to be included into the outpatient reimbursement list / other, please specify<br>If no, then specify
Price reduction of the first biosimilar compared to the reference product	- xxx%
Price reduction of the second, third and further biosimilars	Details of price reduction to be described<br>Or: There is no defined price reduction for subsequent biosimilars.
Price reduction for the reference product	Information on whether a price reduction for the reference product is applied, and if yes, when and to which extent
Any further specificities	e.g., when was it introduced (optional question)
Generic price link	In place: yes / no<br>Details on extent<br>For first and/or subsequent generics<br>For the originator

Source: PPRI 2024

External price referencing

Definition: The practice of using the price(s) of a medicine in one or several countries in order to derive a benchmark or reference price for the purposes of setting or negotiating the price of the product in a given country.

External price referencing is applied for biosimilars.	Yes / no<br>Yes, but only as supplementary policy
If yes, it is applied for the following situations:	For biosimilar medicines in the outpatient and inpatient sectors entering the market / For biosimilar medicines in the outpatient sector entering the market / For biosimilar medicines in the inpatient sector entering the market / For biosimilar medicines aiming to be included into the outpatient reimbursement list / others, please specify
Any specificities	
External price referencing is in place for other medicines than biosimilars.	No / Yes, namely …

Source: PPRI 2024

Tendering

Definition: Any formal and competitive procurement procedure through which tenders (offers) are requested, received, and evaluated for the procurement of goods, works or services, and as a consequence of which an award is made to the tenderer whose tender/offer is the most advantageous.

Some (or all) biosimilars are procured through tendering (or tendering-like) processes.	Yes / no
If yes, the scope with regard to the care sector:	Inpatient sector only / Inpatient and outpatient sectors
**Tendering biosimilars in the inpatient sector**	
Scope of biological medicines tendered	All off-patent biological medicines<br>Several / some, pls. specify:
Institutional framework	Conducted by hospitals / hospital procurement groups / procurement agencies …
Information available on procurement procedures and practices	E.g. regarding award criteria<br>Single vs. multiple-winner tenders
**Tendering biosimilars in the outpatient sector (or tendering-like policies)**	
Scope of biological medicines tendered	All off-patent biological medicines<br>Several / some, pls. specify:
Institutional framework	Conducted at national level by NHS / SHI, by sickness funds
Information available on procurement procedures and practices	E.g. regarding award criteria<br>Single vs. multiple-winner tenders
Generics are tendered (or procured through competitive tendering-like policies).	Inpatient: No / Yes, pls. specify, if needed:<br>Outpatient: No / Yes, pls. specify, if needed:

Source: PPRI 2024; Vogler, Salcher-Konrad and Habimana 2022

4.2 Reimbursement policies 2024

Reference price system

Definition: A reimbursement policy in which identical medicines (ATC 5 level) or similar medicines (ATC 4 level or other groups) are clustered (reference group). The public payer funds a maximum amount (the reference price), while the patient must pay the difference between the reference price and the pharmacy retail price of the medicine, in addition to any co-payments (such as prescription fees or percentage co-payment rates).

Biosimilars are included into the reference price system (RPS) applied in the outpatient sector.	No / yes (in some cases) / no RPS in place
If yes, how many and under which specifications	
If publicly accessible, indicate URL of RPS list	
When were first biosimilars included in the reference price system?	
Any further specificities	
In general, a reference price system is in place (for other medicines than biosimilars), and how those medicines are clustered?	Yes / No<br>ATC 5 cluster / broader clusters than ATC 5

Source: PPRI 2024

Inclusion into reimbursement and co-payments

Definition for reimbursement: Coverage of the cost of reimbursable medicines by a public payer (such as social health insurance / National Health Service). Medicines which may be prescribed at the expense of a public payer are usually included in a **positive list**.

Definition for co-payments: Insured patient's contribution towards the cost of a medicine covered by the third party payer (e.g., a public payer). Common types of co-payments include a percentage of the total cost of the medicine (percentage co-payment), a fixed amount (prescription fee) and a deductible.

**Reimbursement in the outpatient sector**	
Apart from their potential inclusion into the reference price system (see above), biosimilars are included in outpatient reimbursement (positive list).	No / yes (in some cases)
If yes, how many and date of inclusion into reimbursement, if known)	
Extent of reimbursement rates applicable for biosimilars included in the outpatient positive list	xxxx % / not applicable since no percentage reimbursement rates / no percentage co-payments apply)
Any specificities regarding the process of inclusion into outpatient reimbursement compared to other medicines for outpatient use	E.g. shorter timelines, less tightened criteria / committees included, please specify
Indicate URL of outpatient positive list	
Co-payments for biosimilars for outpatient use	Prescription fee, percentage co-payments and deductible
For medicines for outpatient use in general (other medicines than biosimilars)<br>Positive list for medicines in general<br>Percentage reimbursement rates are in place<br>Co-payments for reimbursable outpatient medicines	Positive list / positive and negative list / Negative list only<br>Percentage reimbursement rates are in place / not applied<br>Prescription fee, percentage co-payment and deductible
**Reimbursement in the inpatient sector**	
Biosimilars are included in hospital pharmaceutical formularies (at national, regional and/or facility level	Yes, instead of the originator biological / Yes, additionally to the original biological (and doctors are recommended to switch) / No
Any specificities regarding the process of inclusion into hospital pharmaceutical formularies compared to other medicines for inpatient use	E.g. shorter timelines, less tightened criteria / committees included, please specify
Indicate URLs of hospital pharmaceutical formularies, if publicly accessible	URL of national hospital pharmaceutical formulary:<br>Not publicly available
Co-payments for biosimilars for inpatient use	No
For medicines for inpatient use in general (other medicines than biosimilars):<br>Hospital pharmaceutical formularies<br>Co-payments for medicines for inpatient use	Hospital pharmaceutical formularies at national, regional and/or facility (hospital) level<br>No co-payments for medicines for inpatient use

Source: PPRI 2024

4.3 Specific pricing, procurement and funding policies 2024

For defined medicines, public payers and payers have been piloting and implementing specific funding models (such conditional reimbursement, managed-entry agreements, and subscription models such as the Netflix models). Typically, these funding models apply for high-priced medicines with high uncertainties (e.g., originator biologicals) or for medicines with small populations whose uptake is aimed to be encouraged. The section provides information whether biosimilar medicines are also subject to specific funding models. Furthermore, it also looks into possible anti-trust cases.

Application of special pricing, procurement and reimbursement models for biosimilars (e.g., delinkage / subscription-model, management-entry agreements (MEA))	No / yes (pls. specify cases / medicines and describe details below / No, but under discussion<br>Indicate sector: inpatient / outpatient / inpatient and outpatient sector
Enforcement of anti-trust policies	No / yes (pls. specify and describe details below / No, but under discussion
Specific pricing and funding (e.g., subscription models, MEAs, …) for other medicines	

Source: PPRI 2024

Further information on specific pricing and funding policies and models

Source: indicate source

Further information on anti-trust enforcement:

Source: indicate source

4.4 Supportive tools and practices for policy-makers 2024

Horizon scanning

Definition: The systematic identification of health technologies that are new, emerging or becoming obsolete and that have the potential to affect health, health services and/or society. For the purpose of this study, horizon scanning systems are considered which inform in advance about the loss of exclusivity of originator biologicals and thus support public payers and procurers to be well prepared to harness the benefits of biosimilars from day zero.

Public authorities or public payers use a horizon scanning system to identify loss of exclusivity (patent expiry) of biologics	Yes, systematically / yes, sometimes / yes, but rarely / no
If yes, describe the process and the tools (e.g. regular consultation of a database, horizon scanning scoping meeting with stakeholders), including who use horizon scanning	

Source: PPRI 2024

Health technology assessment

Definition: HTA is a multidisciplinary process that uses explicit methods to determine the value of a health technology at different points in its lifecycle. The purpose is to inform decision-making in order to promote an equitable, efficient, and high-quality health system.

HTA is applied for biosimilar medicines	No / yes (in some cases) / yes, systematically
If yes, in which situations and settings (outpatient and inpatient)	Outpatient / inpatient / outpatient and inpatient<br><br>For biosimilar medicines aiming to be included into the reimbursement list, please specify
Differences in criteria and processes of HTA for biosimilars compared to other medicines	
Any specificities	

Source: PPRI 2024

4.5 Demand-side measures 2024

Definition: Policies that are directed at stakeholders who prescribe (doctors), dispense (pharmacies) or ask for medicines (patients).

Doctors (and further prescribers)

<u>Country context</u>: Only doctors may prescribe / doctors and in a few cases also other health professionals may prescribe, pls. specify

Definitions:

Switch: Action by a prescriber to change medication (e.g. from an originator biological to a biosimilar medicine or from one biosimilar to another biosimilar medicine).

INN prescribing: Requirements for prescribers (e.g. physicians) to prescribe a medicine by its INN, i.e. the active ingredient name instead of the brand name. INN prescribing may be allowed (indicative INN prescribing) or required (mandatory/obligatory INN prescribing).

**Prescribing and switching recommendations**	
Prescribing recommendations for doctors with regard to prescription of biosimilars exist	No / yes
If yes, what is recommended for naïve patients?	Recommendation to prescribe biosimilars for naïve patients / General recommendation to prescribe biosimilars for naïve patients but … / no recommendation on this aspect exists
If yes, what is recommended for patients already on biologic treatment	Recommendation to switch patients for economic reasons / Recommendation that doctors should consider switching under specific conditions, pls. specify / Doctors are asked to decide on a case-by-case basis / Switching is not recommended / Others
Prescription guidelines (including switching guidelines) were issued by	
URL of prescription & switching guidelines	
**Prescribing quota**	
Prescribing quota for doctors with regard to prescription of biosimilars exist	No / yes
If yes, which quota are defined for biosimilars	
Any exemptions	
Any sanctions in case of non-achievement	No / yes, pls specify
Any (financial) incentives for achieving the quota	
Prescribing quota were issued by	
URL of prescribing quota	
Prescribing quota for other non-biosimilar medicines exists	No / yes
**Further gain-sharing models**	
Further gain-sharing models exist (beyond those mentioned above)	No / yes
If yes, pls. describe (care setting: outpatient / inpatient, contract parties, content and incentives of gain-sharing, when was it introduced)	
Publicly accessible information	No / yes, pls. indicate the URL
**INN prescribing**	
In general, prescribing by INN is place (information not focused on biologics)	No, not allowed / yes, mandatory / yes, indicative
If INN prescribing is allowed, there are some exemptions, and biologics are among the exemptions	No exemption for biologicals: biologics and biosimilars may / must be prescribed by INN / Exemptions for biologicals from INN prescribing: biologics and biosimilars must not be prescribed by INN
**Further measures targeted at doctors**	
Information materials for doctors	No / yes, pls. specify (e.g., content, who issued, provided URL)
Information events for doctors (e.g., seminars, trainings)	No / yes, pls. specify
Involvement of doctors in switching studies that evaluate clinical outcomes of a switch	

Source: PPRI 2024

Pharmacists (and further dispensers)

Country context: Only pharmacists may dispense / pharmacists and in a few cases also other health professionals may dispense, pls. specify

Definitions:

Biosimilar substitution: Practice of dispensing a biosimilar medicine instead of the prescribed equivalent and interchangeable biosimilar or biological originator medicine at the pharmacy level without consulting the prescriber.

**Substitution policies**	
(Automatic) substitution for biosimilars	Not, not allowed / Yes, indicative / Yes, mandatory
If yes, for which biologic substances and under which prerequisites?	
If yes, since when, and when was it extended? (process of implementation)	
Any specificities	
URL of underlying law	
Generic substitution in place	No, not allowed /Yes, indicative / Yes, mandatory
**Gain-sharing models**	
Gain-sharing models for pharmacists	No / yes
If yes, pls. describe (care setting: outpatient / inpatient, contract parties, content and incentives of gain-sharing, when was it introduced)	
Publicly accessible information	No / yes, pls. indicate the URL
**Further measures targeted at pharmacists**	
Information materials for pharmacists	No / yes, pls. specify (e.g., content, who issued, provided URL)
Information events for pharmacists (e.g., seminars, trainings)	No / yes, pls. specify

Source: PPRI 2024

Patients

**Information and educational measures**	
Information materials for patients	No / yes, pls. specify (e.g., content, who issued, provided URL)
If yes, provide URL of most common materials	
If yes, key producers of education measures	
Information events for patients (e.g., seminars, trainings)	No / yes, pls. specify
If yes, key providers of educational events	
**Gain-sharing models**	
Gain-sharing models for patients	No / yes
If yes, pls. describe	
Publicly accessible information	No / yes, pls. indicate the URL

Source: PPRI 2024

Annex 3: Methodology of the literature reviews and identified studies

The aim of the literature reviews which supplemented the quantitative data analysis, was to identify evaluation studies investigating whether the implementation of a biosimilar policy, that was in the scope of the study, had impacted the uptake of biosimilars.

This focused research question was explored as part of a more comprehensive literature review with a broader research question, and was conducted in a three-step approach:

- a systematic literature review,
- a targeted narrative literature review, and
- consideration of updates after the finalisation of the literature review.

Systematic literature review

From September until November 2024, a systematic literature review was conducted to explore the body of evidence around biosimilar policies. Based on the findings, studies that analysed the impact of a specific biosimilar policy on biosimilar uptake were selected.

For the larger research question, a study was considered relevant if at least one of the following inclusion criteria was met:

- The study addresses biosimilars,
- The study addresses barriers and facilitators, or challenges or actions to develop or support uptake of biosimilars, and
- The study addresses at least one of the following topics:
  - national and European policy options relating to biosimilars, including policies implemented, abolished, piloted or discussed in the countries included in the study (and beyond if transferable),
  - national biosimilar strategic documents (if applicable),
  - any evidence on the biosimilar knowledge, awareness or hesitancy among different stakeholder groups, and
  - any evidence on the impact of biosimilars with regard to prices, savings and further efficiency gains and overall access to medicines.

The protocol of the systematic literature review was registered in the PROSPERO database on 30 September 2024 (reference number: CRD42024592037).

Searches were conducted in the following databases: Medline via PubMed, Cochrane Library, EMBASE via Ovid.

Only peer-reviewed articles published in English between 1 January 2000 and end of September 2024 were reviewed, with special emphasis on European region.

A total of 8,540 records were identified from the following databases:

- Cochrane (n = 466)
- Embase (n = 3,594)
- PubMed (n = 4,475)
- Other sources (n = 5)

1,951 duplicate records were removed before screening.

6,589 records were screened, and 6,145 records were excluded. Thus, as a next step, 444 records were assessed for eligibility. Thereof, 362 records were excluded, for different reasons:

- Conference abstract only / no full text available (n = 129)
- Not related to the policy or study questions (n = 124)
- Out of the region, policy not transferable (n = 104)
- Not in English (n = 2)
- Duplicate (n = 3)

As a result, 82 studies were selected for inclusion in the comprehensive literature review addressing the broader research questions.

Thereof, **10 studies** reported the outcomes of an evaluation of policy implementation on biosimilar uptake.

- Harsanyi A, Csanadi M, Marky K, Vincziczki ÁZ, Kalo Z, Inotai A. Influence of biosimilar infliximab launch on the utilization pattern of biological medicines: the case of Hungary. Expert Review of Pharmacoeconomics and Outcomes Research. 2020;6(20):653-60
- Hornyák L, Nagy Z, Ilku L, Tálos Z, Endrei D, Ágoston I, et al. Price competition and reimbursement of biosimilar granulocyte-colony stimulating factor in Hungary. Expert Review of Pharmacoeconomics & Outcomes Research. 2019 2019/11/02;19(6):725-31
- Barron A, Chung J, Ferner RE, Leandro M, Maru S, Scourfield A, et al. Effectiveness of biosimilar adoption within a UK tertiary hospital: 6-year follow-up. Br J Clin Pharmacol. 2023;89(10):2944-9
- Moorkens E, Barcina Lacosta T, Vulto AG, Schulz M, Gradl G, Enners S, et al. Learnings from Regional Market Dynamics of Originator and Biosimilar Infliximab and Etanercept in Germany. Pharmaceuticals. 2020;13(10):324. PubMed PMID: doi:10.3390/ph13100324
- Menditto E, Orlando V, Coretti S, Putignano D, Fiorentino D, Ruggeri M. Doctors commitment and long-term effectiveness for cost containment policies: lesson learned from biosimilar drugs. Clinicoecon Outcomes Res. 2015;7:575-81
- Tano M, Paubel P, Ribault M, Degrassat-Théas A. What about offering a financial incentive directly to clinical units to encourage the use of biosimilars? Results of a two-year national experiment in France. Applied Health Economics and Health Policy. 2023 2023/09/01;21(5):799-811
- Tano M, Paubel P, Ribault M, Degrassat-Théas A. Comparison of two financial incentives to encourage the use of adalimumab biosimilars: Results of a french experiment close to clinicians. PharmacoEconomics Open. 2024 2024/11/01/;8(6):897-909
- Jensen TB, Kim SC, Jimenez-Solem E, Bartels D, Christensen HR, Andersen JT. Shift from adalimumab originator to biosimilars in Denmark. JAMA Internal Medicine. 2020;180(6):902-3
- Jensen TB, Bartels D, Sædder EA, Poulsen BK, Andersen SE, Christensen MMH, et al. The Danish model for the quick and safe implementation of infliximab and etanercept biosimilars. European Journal of Clinical Pharmacology. 2020;76(1):35-40
- Vandenplas Y, Simoens S, Van Wilder P, Vulto AG, Huys I. The impact of policy interventions to promote the uptake of biosimilar medicines in Belgium: a nationwide interrupted time series analysis. Health Research Policy and Systems. 2023;21(1):68

Narrative reviews

In the preparation of the country fiches further evaluation of policy implementation on biosimilar uptake identified were included. For this review, we applied broader inclusion criteria (both studies in English and local languages published in the peer-reviewed as well as grey literature).

Through the narrative reviews, we identified four studies in English in the peer-reviewed literature:

- Duggan B, Smith A, Barry M. Uptake of biosimilars for TNF-α inhibitors adalimumab and etanercept following the best-value biological medicine initiative in Ireland. Int J Clin Pharm. 2021;43(5):1251-6
- Attia V, Gaini M, Maugendre E, Pollak C. Biosimilar prescribing incentives: Results of a French pilot of gainsharing between hospitals. Economie et Statistique / Economics and Statistics, Institut National de la Statistique et des Etudes Economiques (INSEE), 2024, (542):147-168
- Oppel S, Renner B, Elsner S, Lippert A. Effects of the introduction of biosimilars on the costs and prescription frequency of selected biologics in statutory and private health insurance in Germany: differences and similarities. Journal of Public Health, 2025. https://doi.org/10.1007/s10389-025-02429-w
- Guidotti E, Vinci B, Attanasio F, Vola F. Effective tools to manage biosimilars prescription: The Italian experience. Health Policy and Technology. 2021;10(1):45-51

Additional studies identified

In addition, we aimed to consider any other eligible literature published up to April 2026. These additional studies were identified through monitoring of more recently published publications, snowballing techniques, and recommendations provided by validators and reviewers.

No further evaluation, which assessed the impact of a policy (package) on biosimilar uptake in a specific setting, was identified.

Two studies published in 2025, which analysed the relationship between biosimilar policies and uptake for some biological substances, across some high-income countries, were also included due to their relevance:

- Tam ACT, Badesha J, Guh DP, Bansback N, Peter KK, Hollis A, et al. Biosimilar Policies and Their Impact on Market Penetration of Adalimumab, Etanercept and Infliximab: A Policy Synthesis and Descriptive Analysis in 13 OECD Countries. BioDrugs. 2025;39(3):461-76
- Shin G, Han H, Choi G, Lee D, Bae S. Demand- Versus Supply-Side Policies in Market Penetration of Biosimilars: Which is More Effective? BioDrugs. 2025;39(4):635-44

Annex 4: Design specifications of biosimilar policies

Biosimilar policies may be implemented through application of different methodologies. The following tables provide background information on specifications in the design of some of the policies presented in Table 2. Where applicable, they also inform whether this policy is also in place for generics (e.g., in cases when biosimilar policies were modelled upon generic policies) in the respective countries.

Additional information on the policies of the biosimilar price link, external price referencing, tendering, and tendering-like practices in 27 European countries, 2024

Policy	Biosimilar price link		EPR for biosimilars		Tendering			Tendering-like practices for Bx
	In place	Scope of medicines[1]	Applied for Bx	For new medicines	Applied for Bx	Framework	Procedures[2]	
Austria	Yes	Bx & RP	No	Yes	Yes	Decentr.	Mainly price<br>Multi-winner	No
Belgium	Yes	Bx & RP	No	Yes	Yes	Decentr.	MEAT	No
Bulgaria	Yes	Bx & RP	Yes	Yes	Yes	Decentr.	Price<br>Single-winner	No
Croatia	Yes	Only Bx	Yes	Yes	Yes	Central. & decentr.	Price<br>Single-winner	No
Czechia	Yes	Only Bx	Yes	Yes	Yes	Decentr.	MEAT<br>Multi-winner	Yes, by health insurances
Estonia	Yes	Only Bx	No	Yes	Yes	Central. & decentr.	Price<br>Single-winner	No
Finland	Yes	Only Bx	Yes	Yes	Yes	Region.	Mainly price<br>Mainly single-winner	No
France	Yes	Bx & RP	Yes	Yes	Yes	Central. & region.	Price & MEAT<br>Mainly single-winner	No
Germany	No	N. appl.	No	Yes[3]	Yes	Decentr.	N.a.	Yes, by sickness funds
Greece	No	N. appl.	Yes	Yes	Yes	Central. & decentr.	MEAT<br>Multi-winner	No
Hungary	Yes	Only Bx	Yes	Yes	Yes	Central. & decentr.	MEAT<br>Single & multi-winner	Yes, by social insurance
Ireland	Yes	Bx & RP	No	Yes	Yes	Decentr.	Price<br>Single winner	No
Italy	Yes	Only Bx	No	Yes	Yes	Central. & region.	MEAT<br>Multi-winner[4]	Yes, by regions
Latvia	Yes	Only Bx	No	Yes	Yes	Decentr	MEAT<br>Single & multi-winner	No
Lithuania	Yes	Only Bx	Yes	Yes	Yes	Central. & decentr.	MEAT<br>Single-winner	No
Luxembourg	No	N. appl.	Yes	Yes	Yes	Decentr.	N.a.	No
Netherlands	No	N. appl.	No	Yes	Yes	Decentr.	Price only	Yes, by health insurers
Norway	Yes	Bx & RP	Yes	Yes	Yes	Central.	MEAT<br>Multi-winner	No
Poland	Yes	Only Bx	Yes	Yes	Yes	Central. & decentr.	Price<br>Single-winner	No
Portugal	Yes	Only Bx	Yes	Yes	Yes	Central. & decentr.	Price & MEAT<br>Single- & multi-winner	No
Romania	Yes	Only Bx	Yes	Yes	Yes	Central. & decentr.	MEAT	Yes, by social insurance
Slovakia	Yes	Only Bx	Yes	Yes	Yes	Central. & decentr.	Price	Yes, by health insurers
Slovenia	Yes	Only Bx	Yes	Yes	Yes	Decentr.	MEAT<br>Multi-winner	No
Spain	Yes	Only Bx	Yes	Yes	Yes	Region.	MEAT<br>Multi-winner	No
Sweden	No	N. appl.	No	No	Yes	Region.	Mostly price<br>Single- & multi-winner	No
Switzerland	Yes	Only Bx	No	Yes	Yes	Decentr.	N.a.	No
United Kingdom	No	N. appl.	No	No	Yes	Central.	MEAT	No

Definitions of the policies are provided in Table 1 in the main body of the manuscript. Information on the implementation of the biosimilar policies, and design characteristics was sourced from the Pharmaceutical Pricing and Reimbursement Information (PPRI) policy network of public authorities responsible for pharmaceutical pricing and reimbursement and validated by experts. [1] Can apply solely for biosimilars (requirement to reduce the price in relation to the reference product) or may also concern the reference product (RP) which may be requested to reduce the price upon market entry or inclusion of the biosimilar in the reimbursement list, or a defined time period after. [2] Information on commonly applied award criteria for tendering (e.g., price only, or application of Most Economically Advantageous Tender (MEAT) criteria, and on the relevance of multiple-winner tender. [3] As a supplementary policy, abolished in 2025. [4] Obligation for multiple-winner tender contracts in cases of three or more biosimilars on the market. *Abbreviations*: Bx: biosimilars. Central.: centralised procurement (i.e. through national procurement agency for hospitals, the ministry of health or a social health insurance fund). Decentr.: decentralised procurement (i.e. tendering done by hospitals and hospital groups). MEAT: Most Economically Advantageous Tender (use of further award criteria in addition to the price). N.a.: not available. N. appl.: not applicable. Region.: regional procurement, RP: Reference product.

Additional information on the policies of the reference price system, horizon scanning, and International Non-Proprietary Name (INN) for biologicals in 27 European countries, 2024

Policy	Reference price system		Horizon scanning on Loss of Exclusivity	INN prescribing		Switching[1]	
	Incl. biosimilars	In general		For biologicals	In general	In place	Design
Austria	No	No	Yes, done occasionally	No	No	Yes	Decision on a case-by-case basis, for economic reasons
Belgium	Yes	Yes	Yes, done occasionally	Yes	Yes	Yes	Recommended for economic reasons
Bulgaria	Yes	Yes	No	Yes	Yes	No	No specific recommendation
Croatia	Yes	Yes	No	Yes	Yes	Yes	Decision on a case-by-case basis
Czechia	Yes	Yes	No	Yes	Yes	Yes	Recommended for economic reasons
Estonia	Yes	Yes	Yes, done occasionally	No	Yes	Yes	Recommended for economic reasons, Physicians have to “opt-out” and justify, if they do not switch
Finland	Yes	Yes	No	Yes	Yes	Yes	Mandatory biosimilar prescribing and switching to the lowest price biological when renewing the prescription (prescription is valid one year)
France	No	Yes	Yes, done systematically	No	Yes	Yes	Recommended for economic reasons
Germany	Yes	Yes	No	No	Yes	Yes	Recommended for economic reasons. Physicians and patients can opt out
Greece	Yes	Yes	No	No	Yes	Yes	Recommended for economic reasons. Physicians and patients can opt out
Hungary	Yes	Yes	Yes, done systematically	Yes	Yes	Yes	Decision on a case-by-case basis
Ireland	No	Yes	Yes, done occasionally	Yes	Yes	Yes	Recommended
Italy	No	Yes	Yes, done systematically	Yes	Yes	Yes	Recommended, but doctors are asked to decide on a case-by-case basis
Latvia	Yes	Yes	No	Yes	Yes	Yes	Recommended for economic reasons
Lithuania	Yes	Yes	Yes, done occasionally	Yes	Yes	Yes	Recommended for economic reasons
Luxembourg	No	No	No	No	No	N.a.	N.a.
Netherlands	Yes	Yes	Yes, done systematically	Yes	Yes	Yes	Recommended under defined conditions (e.g., supervision)
Norway	Yes	Yes	Yes, done systematically	Yes	Yes	Yes	Recommended for economic reasons
Poland	Yes	Yes	Yes, done systematically	Yes	Yes	Yes	No specific recommendation
Portugal	No	Yes	Yes, done occasionally	Yes	Yes	Yes	Recommended for economic reasons
Romania	Yes	Yes	No	No	Yes	Yes	Recommended, doctors should consider switching within 12 months after a biosimilar medicine was added to the reimbursement (reference price) list
Slovakia	Yes	Yes	No	Yes	Yes	Yes	Recommended for economic reasons
Slovenia	Yes	Yes	No	Yes	Yes	Yes	Recommendation that doctors should consider switching under specific conditions
Spain	Yes	Yes	Yes, done occasionally	No	Yes	Yes	Recommended for economic reasons
Sweden	No	No	Yes, done occasionally	No	No	Yes	Decision on a case-by-case basis. Multiple switching is not recommended. Managed-switch programs for biologics have been implemented at the regional level (e.g. for infliximab in the region of Skane)
Switzerland	No	Yes	Yes, done occasionally	Yes	Yes	No	No specific recommendation

Policy	Reference price system		Horizon scanning on Loss of Exclusivity	INN prescribing		Switching[1]	
	Incl. biosimilars	In general		For biologicals	In general	In place	Design
United Kingdom	No	No	Yes, done systematically	No	Yes	Yes	Doctors are asked to decide on a case-by-case basis

Definitions of the policies are provided in Table 1 in the main body of the manuscript. Information on the implementation of the biosimilar policies, and design characteristics was sourced from the Pharmaceutical Pricing and Reimbursement Information (PPRI) policy network of public authorities responsible for pharmaceutical pricing and reimbursement and validated by experts.
[1] Relates to a situation with a policy in place that obliges, encourages or supports switching to a biosimilar by prescribers (e.g., regulatory guidance document). *Abbreviations*: Bx: biosimilars. INN: International Non-Proprietary Name. N.a.: not available.

Additional information on the policies of prescribing quotas, gain-sharing and biosimilar substitution in 27 European countries, 2024

Policy	Prescribing quotas		Gain-sharing		Biosimilar substitution[2]	
	In place for Bx	Design	In place for Bx	Design	In place for Bx	Design
Austria	No	N. appl.	No	N. appl.	No	N. appl.
Belgium	Yes	For selected biological substances in the outpatient sector	Yes	In the inpatient sector	No	N. appl.
Bulgaria	No	N. appl.	No	N. appl.	Yes	Indicative biosimilar substitution in community pharmacies for all biological substances
Croatia	Yes	Prescribing quotas not specified	No	N. appl.	No	N. appl.
Czechia	No	N. appl.	No	N. appl.	Yes	Indicative biosimilar substitution in community pharmacies for all biological substances
Estonia	No	N. appl.	No	N. appl.	Yes	Mandatory biosimilar substitution in community pharmacies for all biological substances
Finland	No	N. appl.	No	N. appl.	Yes	Mandatory biosimilar substitution in community pharmacies for defined biological substances, implemented in a step-wise approach
France	Yes	For selected biological substances in inpatient and outpatient settings	Yes	In outpatient sector for defined biological substances	Yes	Indicative biosimilar substitution in community pharmacies for defined biological substances
Germany	Yes	For selected biological substances and variation across regions and sickness funds	Yes	For selected biological substances and variation across regions and sickness funds	Yes	Indicative biosimilar substitution in community pharmacies for some defined biological substances
Greece	Yes	For selected biological substances in inpatient and outpatient settings	No	N. appl.	No	N. appl.
Hungary	Yes	For selected biological substances in inpatient and outpatient settings	Yes	In inpatient and outpatient sectors	No	N. appl.
Ireland	No	N. appl.	No[1]	N. appl.	No	N. appl.
Italy	Yes	Regional quotas with variations in product scope	Yes	Regional models for defined biological substances	No	N. appl.
Latvia	Yes	Prescribing quotas not specified	No	n.a.	Yes	Indicative biosimilar substitution
Lithuania	No	N. appl.	No	N. appl.	No	N. appl.
Luxembourg	N.a.	N. appl.	No	N. appl.	No	N. appl.
Netherlands	Yes	Prescribing quotas not specified	Yes	Fixed clawbacks for all biosimilars	Yes	Indicative biosimilar substitution for all biological substances
Norway	No	N. appl.	No	N. appl.	Yes	Indicative biosimilar substitution for defined biological substances, implemented in a step-wise approach

Policy	Prescribing quotas		Gain-sharing		Biosimilar substitution[2]	
	In place for Bx	Design	In place for Bx	Design	In place for Bx	Design
Poland	No	N. appl.	No	N. appl.	Yes	Indicative biosimilar substitution for all biological substances
Portugal	Yes	Prescribing quotas for selected biological substances in the inpatient sector	Yes	In inpatient sector	No	N. appl.
Romania	Yes	For all biological substances	No	N. appl.	No	N. appl.
Slovakia	Yes	For all biological substances for newly diagnosed patients	No	N. appl.	No	N. appl.
Slovenia	No	N. appl.	No	N. appl.	No	N. appl.
Spain	Yes	Prescribing quotas not specified	Yes	Regional models for defined biological substances	No	N. appl.
Sweden	Yes	Prescribing quotas not specified	Yes	Regional models in inpatient sector for defined biological substances	No	N. appl.
Switzerland	No	N. appl.	No	N. appl.	Yes	Indicative biosimilar substitution for all biological substances
United Kingdom	Yes	Specific targets for newly diagnosed patients	Yes	Primarily in inpatient sector, but also outpatient	No	N. appl.

Definitions of the policies are provided in Table 1 in the main body of the manuscript. Information on the implementation of the biosimilar policies, and design characteristics was sourced from the Pharmaceutical Pricing and Reimbursement Information (PPRI) policy network of public authorities responsible for pharmaceutical pricing and reimbursement and validated by experts.
[1] No gain-sharing in 2024, but some pilots in earlier times. [2] Relates to biosimilar substitution in community pharmacy. *Abbreviations*: Bx: biosimilars. N.a.: not available. N. appl.: not applicable.

Annex 5: Description of pieces in the literature that evaluated impact of biosimilar policies on biosimilar uptake

Policy / policies	Country / countries	Setting (if applicable)	Substances	Uptake	Description	Study
**Individual policy**						
Biosimilar price link	Hungary	-	Infliximab	No increase	This study assessed the impact of the biosimilar price link policy on infliximab uptake in Hungary. Despite biosimilars being 30% lower priced than the reference product, this did not result in increased use of infliximab. The authors considered the following barriers as a contributing factor to the study findings: lack of policy support (e.g., no mandatory switching), slow procurement processes (6-month delay in biosimilar uptake after approval), and physician reluctance due to perceived safety and efficacy concerns.	Harsanyi et al. 2020[1]
Tendering-like system	Hungary	Outpatient	Granulocyte Colony-Stimulating Factors (G-CSFs)	Increase	This study analysed the impact of tendering by the social health insurance on public spending and biosimilar uptake in Hungary. Medicines with the lowest or near-lowest bids received preferred status, full reimbursement, and minimal co-payment, while higher-priced options incurred greater patient costs. Following two tender rounds for Granulocyte Colony-Stimulating Factors (G-CSFs), public spending dropped by 44% in the first year and 7.9% in the second. Despite stable overall patient numbers, biosimilar uptake rose sharply (+130.4% and +147.3%).	Hornyák et al. 2019[2]
Encouraging switching (support by a Biosimilar Steering Group)	United Kingdom	Hospital	Several (including adalimumab, etanercept, infliximab, and rituximab)	Increase	This study assessed the impact of a hospital-based Biosimilar Steering Group (BSG) in facilitating switching protocols and biosimilar uptake in the UK. The BSG provided centralised oversight for switching protocols, reviewed patient-specific exemption and reversion requests, and maintained a governance framework across specialties since it operates at a hospital level. This coordinated approach enabled a faster and more complete transition to biosimilars, outperforming national and regional National Health Service (NHS) averages in biosimilar uptake. As a result, this approach realised additional cost savings of approximately £ 380,000 in 2021/2022 alone.	Barron et al. 2023[3]
Prescribing quotas	Germany	Outpatient, different regions	Infliximab, etanercept	Increase	Biosimilar uptake improved in German regions where regional sickness funds had implemented prescribing quotas alongside with more rigorous monitoring mechanisms, compared to regions where this was not the case.	Moorkens et al. 2020[4]
Prescribing quotas	Italy	One region (Campania)	Not specified	Mixed, initially increase	This study analysed long-term effectiveness of prescription quotas on biosimilar uptake in Campania region of Italy. The policy began with Decree 15 (November 2009), requiring all prescribers to prefer biosimilars with equal therapeutic value and administration regimen and targeting at least 40% savings per therapy. Decree 44 (July 2010) clarified this applied to naïve patients. Decree 34 (March 2012) stated the lowest-cost biosimilar should be preferred for naïve patients. Decree 27 (March 2013) mandated the lowest-cost biosimilar as the first-line treatment option in naïve patients unless clinically justified otherwise. Biosimilar uptake increased from 1.6% (2009) to 35.9% (2013) for all biological medicines, with a compound annual growth rate (CAGR) of 107.2%, compared to 68.8% nationally. After 2012, biosimilar uptake plateaued, and originator medicines use started to increase again, reversing earlier savings.	Menditto et al. 2015[5]

Policy / policies	Country / countries	Setting (if applicable)	Substances	Uptake	Description	Study
Gain-sharing	France	Hospital	Etanercept	Increase	An evaluation showed that one of the models, which redirected savings to the wards that were responsible for biosimilar prescribing, instead of offering the savings of the hospitals in general, had higher impacts.<br>This study analysed impact of gain-sharing (with direct financial incentives to clinical units) on etanercept biosimilar prescriptions in French hospitals. In the general case, 20% of the price difference between the reference product and its biosimilar was redirected to hospitals for each biosimilar dispensed in community pharmacies based on hospital prescriptions. In contrast, under the experimental case, 30% of this difference was allocated, with a mandatory share going directly to the prescribing clinical units. At the start of the experiment, there was an acceleration of biosimilar penetration in the experimental group, until both groups reached a plateau. A significant double difference estimator of 9.72 percentage points in favour of the experiment confirmed this. The authors concluded that incentivising prescribers directly (through their clinical units) was significantly more effective in increasing biosimilar uptake than incentives given to hospitals generally.	Tano et al. 2023[6]
Gain-sharing	France	Hospital	Adalimumab	Increase	This follow-up study on Tano et al. 2023 assessed the extension of gain-sharing to adalimumab, analysing uptake differences based on prior participation in similar initiatives. General case hospitals had a significantly lower mean biosimilar uptake (16.7% vs. 23.2%, p=0.029) than those included in the experimental case. Twenty-three of the 40 hospitals in the experimental case and ten out of the 91 hospitals studied in the general case had already taken part in the etanercept experiment. The authors concluded that prior involvement in similar incentive schemes may encourage prescribers to adopt biosimilars in subsequent programs, although this effect was limited in statistical power.	Tano et al. 2024[7]
Gain-sharing	France	Hospital prescribing supplied in community pharmacies	Insulin glargine, etanercept	Increase	A gain-sharing model was piloted for hospital prescribing of medicines (insulin glargine and etanercept) supplied in community pharmacies in France which provided for sharing of the gains between the hospitals and the social insurance, and incentives were redistributed to the prescribing units. During the pilot between October 2018 and September 2021, biosimilar use increased for both insulin glargine (+6.0 percentage points) and etanercept (+10.8 ppt). The pilot generated 0.5% cost savings for insulin glargine and 0.1% for etanercept.	Attia et al. 2024[8]
Gain-sharing	Ireland	Hospital	Adalimumab, etanercept	Increase	In June 2019, Ireland's national health authority, the Health Service Executive (HSE), launched a gain-sharing initiative as part of its "best-value biological" medicine initiative targeting adalimumab and etanercept. The program, which ran from 2019 to 2021, designated specific biosimilar versions of these biologics as "best-value" options. To encourage their use, the HSE set a prescription target of 80% for these best-value biologics combined y a financial incentive scheme. Under the gain-sharing program, hospital departments received € 500 for each patient who was either newly prescribed or switched to a designated best-value biologic. After one year, the uptake of these biologics reached 50%, encompassing nearly 12,000 patients and generating savings of € 22 million.	Duggan et al. 2021[9]

Policy / policies	Country / countries	Setting (if applicable)	Substances	Uptake	Description	Study
**Mix of policies**						
Switching guidelines, communication to healthcare professionals and patients and tendering	Denmark	Hospital	Adalimumab	Increase	This study analysed the impact of a centralised tendering on biosimilar uptake and cost savings in Denmark. After the originator's patent expired in October 2018, three biosimilars were selected: one for paediatrics and two for adults. Uptake increased from 71.6% in November to 95.1% in December 2018. Costs dropped by 82.8% in the same period. In December, biosimilar use reached 77.9% in paediatrics, 94.7% in Western Denmark adults, and 97.7% in Eastern Denmark adults. Uptake was consistent across specialties, including dermatology, gastroenterology, and rheumatology.	Jensen et al. 2020a[10]
Switching guidance, communication to healthcare professionals and patients, and tendering	Denmark	Hospital sector	Etanercept, infliximab	Increase	This study evaluated Denmark's rapid biosimilar uptake following centralised tendering and awareness campaigns for infliximab and etanercept. Rapid shift from originator infliximab to its biosimilar was achieved following the patent expiry in February 2015. The first sale of a biosimilar to a hospital department took place in March 2015, by April it constituted 18.9% of the total infliximab in Defined Daily Doses (DDD) and went up to 90.6% in July. The cost of infliximab was reduced by two-thirds when switching to biosimilar, equivalent to a cost saving of DKK 200 million (approx. £ 24 million). The shift from etanercept originator to biosimilar showed similar pattern. The first sales of etanercept biosimilar to a hospital department took place in April 2016 (biosimilar had 15.3% of total etanercept consumption) and reached 85.4% in June 2016. The fast uptake of biosimilars was attributed to centralised tendering and educational and awareness-raising activities.	Jensen et al. 2020b[11]
Prescribing quotas, inclusion in the reference price system, and design of pricing policies	Germany	Outpatient, statutory vs. private health insurance	Adalimumab, infliximab, rituximab, and trastuzumab	Partially increase	The study examined the uptake of biosimilars across statutory and private health insurance settings in Germany. Their findings indicate that the statutory health insurance system, which is characterized by a broader application of policies such as prescribing quotas and the inclusion of biosimilars in the reference price system, demonstrated more pronounced shifts from originator biologicals to biosimilars.	Oppel et al. 2025[12]
Prescription quotas, financial incentive, monitoring on adequate hospital tendering, education campaign (two waves of policy interventions)	Belgium	Outpatient and hospital	Etanercept (outpatient), filgrastim (hospital), and epoetin (hospital)	Mixed	This study evaluated the impact of prescription quotas, tendering (monitoring) and education campaigns on biosimilar uptake. The 2016 intervention introduced 20% prescription targets for naïve patients and hospital tender monitoring. The 2019 intervention added financial incentives and an information campaign. Results showed mixed effects: small changes in epoetin and filgrastim uptake post-2016, a decrease in outpatient etanercept use after 2019 incentives, and significant uptake increases for epoetin and filgrastim in hospitals following the campaign.	Vandenplas et al. 2023[13]

Policy / policies	Country / countries	Setting (if applicable)	Substances	Uptake	Description	Study
Mandatory regional tendering, communication, and financial incentive	Italy	Three regions (Tuscany, Friuli Venezia Giulia, Veneto)	Etanercept, epoetin, rituximab, and somatropin	Increase	An analysis of biosimilar market shares for four substances across three Italian regions revealed that Tuscany achieved the highest uptake. The positive developments in Tuscany were attributed to a comprehensive, three-pillar strategy: 1) A regional resolution (Resolution n °194/2018, dated 26 February 2018) on biosimilar prescribing and use mandated that Tuscan healthcare organisations prescribe biological products awarded through regional tenders. If the awarded product was a biosimilar, physicians were expected to prescribe it. In cases where the tendered product was an originator biological, prescribing required evaluation and approval by the regional authority. 2) An engagement with the managers and specialists of the Tuscan local health authorities and teaching hospitals to jointly define targets for biosimilar uptake. This was supported by bi-monthly meetings of chief medical officers to review uptake rates and associated savings. 3) Development of a pay-for-performance system.	Giudotti et al. 2021[14]
**Single policies across countries**						
Tendering, biosimilar price link, and prescribing quotas	13 countries (including 9 European countries, see description)	Outpatient and hospital	Adalimumab, etanercept, and infliximab	Increase	The study aimed to examine biosimilar uptake and expenditure for three Tumour Necrosis Factor (TNF) inhibitor biological substances in 13 countries (Australia, Belgium, Canada, France, Germany, Italy, Japan, South Korea, Norway, Spain, Sweden, Switzerland, and the United Kingdom). Thereby, two countries for a given setting (retail or hospital) and originator were paired if they differed only on one specific policy, which resulted in the analysis of three policies: tendering, biosimilar price link and prescribing quotas.<br>IQVIA MIDAS sales data from 2012 to 2023 were analysed. The study findings suggest that the three policies appeared to facilitate greater biosimilar uptake, but this did not consistently translate to expenditure reductions. Solely tendering appeared to achieve expenditure reductions.	Tam et al. 2025[15]
Five demand-side measures (financial incentives, prescribing guidelines, prescription budgets, prescription quotas and education and information initiatives) and four supply-side measures (price link, tendering, internal and external price referencing)	8 countries (including 6 European countries, see description)	Not specific	Infliximab, rituximab, and trastuzumab	Stronger increase due to demand-side measures	The study aimed to evaluate the impact of different demand- and supply-side policies on biosimilar uptake (measured through two indicators: biosimilar market shares and the time required for biosimilars to reach 50% market share. IQVIA MIDAS sales data from July 2012 to June 2020 were analysed for ten countries, thereof six European countries (France, Germany, Italy, Spain, Sweden, and the United Kingdom).<br>The study showed that demand-side policies, such as financial incentives and prescribing guidelines, are significantly associated with higher biosimilar market share and shorter time to reach market saturation.	Shin et al. 2025[16]

References

1. Harsanyi A, Csanadi M, Marky K, Vincziczki ÁZ, Kalo Z, Inotai A. Influence of biosimilar infliximab launch on the utilization pattern of biological medicines: the case of Hungary. Expert Review Of Pharmacoeconomics And Outcomes Research. 2020;6(20):653-60.

2. Hornyák L, Nagy Z, Ilku L, Tálos Z, Endrei D, Ágoston I, et al. Price competition and reimbursement of biosimilar granulocyte-colony stimulating factor in Hungary. Expert Review of Pharmacoeconomics & Outcomes Research. 2019;19(6):725-31.
3. Barron A, Chung J, Ferner RE, Leandro M, Maru S, Scourfield A, et al. Effectiveness of biosimilar adoption within a UK tertiary hospital: 6-year follow-up. Br J Clin Pharmacol. 2023;89(10):2944-9.
4. Moorkens E, Barcina Lacosta T, Vulto AG, Schulz M, Gradl G, Enners S, et al. Learnings from Regional Market Dynamics of Originator and Biosimilar Infliximab and Etanercept in Germany. Pharmaceuticals. 2020;13(10):324.
5. Menditto E, Orlando V, Coretti S, Putignano D, Fiorentino D, Ruggeri M. Doctors commitment and long-term effectiveness for cost containment policies: lesson learned from biosimilar drugs. Clinicoecon Outcomes Res. 2015;7:575-81.
6. Tano M, Paubel P, Ribault M, Degrassat-Théas A. What about offering a financial incentive directly to clinical units to encourage the use of biosimilars? Results of a two-year national experiment in France. Applied Health Economics and Health Policy. 2023;21(5):799-811.
7. Tano M, Paubel P, Ribault M, Degrassat-Théas A. Comparison of two financial incentives to encourage the use of adalimumab biosimilars: Results of a french experiment close to clinicians. PharmacoEconomics Open. 2024;8(6):897-909..
8. Attia V, Gaini M, Maugendre E, Pollak C. Biosimilar prescribing incentives: Results of a French pilot of gainsharing between hospitals. Economie et Statistique / Economics and Statistics, Institut National de la Statistique et des Etudes Econo-miques (INSEE). 2024 (542):147-68.
9. Duggan B, Smith A, Barry M. Uptake of biosimilars for TNF-α inhibitors adalimumab and etanercept following the best-value biological medicine initiative in Ireland. Int J Clin Pharm. 2021;43(5):1251-6.
10. Jensen TB, Kim SC, Jimenez-Solem E, Bartels D, Christensen HR, Andersen JT. Shift from adalimumab originator to biosimilars in Denmark. JAMA Internal Medicine. 2020;180(6):902-3.
11. Jensen TB, Bartels D, Sædder EA, Poulsen BK, Andersen SE, Christensen MMH, et al. The Danish model for the quick and safe implementation of infliximab and etanercept biosimilars. European Journal of Clinical Pharmacology. 2020;76(1):35-40.
12. Oppel S, Renner B, Elsner S, Lippert A. Effects of the introduction of biosimilars on the costs and prescription frequency of selected biologics in statutory and private health insurance in Germany: differences and similarities. Journal of Public Health. 2025. https://doi.org/10.1007/s10389-025-02429-w.
13. Vandenplas Y, Simoens S, Van Wilder P, Vulto AG, Huys I. The impact of policy interventions to promote the uptake of biosimilar medicines in Belgium: a nationwide interrupted time series analysis. Health Research Policy and Systems. 2023;21(1):68.
14. Guidotti E, Vinci B, Attanasio F, Vola F. Effective tools to manage biosimilars prescription: The Italian experience. Health Policy and Technology. 2021;10(1):45-51.
15. Tam ACT, Badesha J, Guh DP, Bansback N, Peter KK, Hollis A, et al. Biosimilar Policies and Their Impact on Market Penetration of Adalimumab, Etanercept and Infliximab: A Policy Synthesis and Descriptive Analysis in 13 OECD Countries. BioDrugs. 2025;39(3):461-76.
16. Shin G, Han H, Choi G, Lee D, Bae S. Demand- Versus Supply-Side Policies in Market Penetration of Biosimilars: Which is More Effective? BioDrugs. 2025;39(4):635-44.
